# JK5G postbiotics modulate gut microbiota and metabolome to alleviate cancer-related pain: a randomized controlled trial with multi-omics integration

**DOI:** 10.3389/fimmu.2026.1764491

**Published:** 2026-03-04

**Authors:** Mengting Chen, Junhui Zhang, Hong Yang, Lei Lei, Liejun Yang, Sixiong Wang, Huiqing Yu

**Affiliations:** 1 Department of Clinical Nutrition, Chongqing University Cancer Hospital, School of Medicine, Chongqing University, Chongqing, China; 2 Department of Geriatric Oncology, Chongqing University Cancer Hospital, School of Medicine, Chongqing University, Chongqing, China; 3 Department of Palliative Care, Chongqing University Cancer Hospital, School of Medicine, Chongqing University, Chongqing, China

**Keywords:** cancer pain, gut microbiota, machine learning, metabolomics, postbiotics, SHAP

## Abstract

**Introduction:**

Cancer-related pain remains a critical clinical challenge, with existing opioid-based therapies often yielding inadequate relief and significant side effects. This study investigates the therapeutic potential of JK5G postbiotics—a formulation of inactivated *Lactobacillus* strains and metabolites—in modulating the gut-microbiome-immune axis to alleviate pain in cancer patients.

**Methods:**

This study employs a randomized, double-blind, placebo-controlled trial design involving 149 participants divided into two groups: a control group receiving patient-controlled subcutaneous analgesia (PCSA) plus placebo, and an experimental group receiving PCSA plus JK5G postbiotics. The primary outcomes were changes in gut microbiota composition assessed by 16S rRNA gene sequencing, and quality of life (QoL). The secondary outcomes included fecal metabolomics, adverse effects (AEs), blood inflammatory cytokines, and lymphocyte subsets. This study was registered at www.chictr.org.cn**(ChiCTR2500108811).**

**Results:**

JK5G supplementation significantly improved pain scores, QoL, and cognitive and social functioning compared to controls. Microbiome analysis revealed enrichment of beneficial taxa such as *Akkermansia muciniphila* and *Bifidobacterium*, alongside suppression of pathogenic *Escherichia-Shigella*. Machine learning identified five core microbial biomarkers (*Akkermansia muciniphila*, *Bifidobacterium*, *Escherichia-Shigella*, *Blautia*, *Streptococcus*), with SHAP analysis highlighting *Akkermansia muciniphila* and *Bifidobacterium* as top contributors. Metabolomic profiling demonstrated upregulation of 236 metabolites, including kynurenic acid and butyric acid, with tryptophan and butyrate metabolism emerging as key altered pathways. Immune profiling showed elevated CD3^+^CD4^+^ T cells and reduced TNF-α levels, while MIMOSA2 analysis linked microbial taxa to metabolic shifts, such as correlations between *Ruminococcus torques* and butyric acid.

**Conclusion:**

These findings suggest that JK5G may contribute to the amelioration of cancer-related pain by reshaping gut microbiota, modulating host metabolism, and enhancing immune responses. This study highlights the potential of JK5G postbiotics as an adjunct therapy, supporting the need for further validation in larger cohorts and mechanistic investigations to advance its clinical translation.

**Clinical Trial Registration:**

https://www.chictr.org.cn/showproj.html?proj=285304, identifier ChiCTR2500108811.

## 1 Introduction

Cancer pain, particularly in its moderate to severe forms, remains one of the most prevalent and debilitating complications among patients with malignancies worldwide (1). This symptom is not only persistent but also profoundly impairs patients’ daily functioning, psychological well-being, and overall quality of life (QoL), often overshadowing the survival benefits of anti-cancer therapies (2). Epidemiological studies indicate that more than half of individuals undergoing active cancer treatment, and an even greater proportion of those with advanced disease, experience significant pain symptoms (1). Such pain is frequently under-recognized and undertreated, especially in populations with complex comorbidities or those receiving palliative care (3, 4). The social and economic ramifications of inadequately controlled cancer pain extend beyond the individual, affecting families and healthcare systems through increased care needs, loss of productivity, and heightened medical expenditures (4, 5).

Despite advances in oncologic therapeutics and palliative care, the management of moderate to severe cancer pain continues to rely predominantly on opioid analgesics, in accordance with the World Health Organization’s and National Comprehensive Cancer Network (NCCN) recommendations of the analgesic ladder as the clinical cornerstone (6). While opioids such as morphine and hydromorphone have demonstrated efficacy in pain alleviation, their use is commonly associated with a spectrum of adverse effects (AEs), including constipation, nausea, sedation, respiratory depression, and the risk of dependency. These adverse effects can limit patient compliance and compromise QoL (6). Moreover, because of inter-individual variability in opioid responsiveness and tolerability, frequent dose adjustments and the adoption of adjunctive or alternative pain management strategies are often necessary (7). Multidisciplinary interventions and non-pharmacologic modalities, such as acupuncture or minimally invasive procedures, have shown some promise in enhancing analgesic outcomes and mitigating opioid-related toxicity; however, these approaches are not universally accessible, nor are they consistently effective (8). The unmet need for more targeted, well-tolerated, and integrative pain management options is thus increasingly recognized in oncology care paradigms.

In recent years, the role of the gut microbiota and its metabolites in modulating pain, inflammation, and host immune function has garnered significant attention. Perturbations of the intestinal microbial ecosystem, known as dysbiosis, are increasingly recognized for their potential influence on the pathogenesis and persistence of cancer-related pain through complex interactions with the immune and neuroendocrine systems (9, 10). In this context, immunonutrition—targeted nutritional strategies designed to modulate immune function—has emerged as a promising adjuvant in cancer care (11). By acting on the gut-immune axis, dietary and microbial interventions can regulate systemic inflammation and potentially alleviate cancer-related symptoms, including pain (10).

Novel therapeutic strategies targeting the gut microbiome, such as the administration of probiotics, prebiotics, and, more recently, postbiotics, are being actively investigated. Postbiotics, defined as non-viable microbial cells, their metabolites, and cell components, exert immunomodulatory and health-promoting effects, thereby functioning within the conceptual framework of immunonutrition (12). They directly interact with the gut microbiota and host immunity while avoiding the risks associated with live probiotics. Specific postbiotics, such as JK5G—comprising inactivated lactic acid bacteria and their bioactive metabolites—have demonstrated the ability to restore microbial balance, attenuate inflammatory cytokine production, and improve nutritional and immune status in patients with advanced cancer (12).

Despite these promising findings, high-quality clinical data specifically addressing the impact of postbiotics on cancer pain, as well as data on the integration of postbiotics with standard analgesic regimens, remain limited. More importantly, there is a paucity of evidence from integrated, multi-omics clinical investigations that simultaneously delineate how postbiotic-induced modulation of the gut microbiome translates into changes in host metabolism and systemic immunity, ultimately affecting pain perception and QoL in cancer patients (12). This critical knowledge gap underscores the need for rigorous clinical trials that not only evaluate the analgesic efficacy of postbiotics but also elucidate their mechanism of action within the gut-microbiome-immune axis—a core target of immunonutrition. Such studies are essential to advance the development of targeted, microbiome-based adjuvant therapies for cancer pain management.

To address this critical gap, we conducted a randomized, double-blind, placebo-controlled trial. The study aimed to evaluate whether adjunctive supplementation with the specific postbiotic JK5G, combined with a standardized nutritional plan to control for dietary confounders, could enhance pain management and QoL in patients with moderate to severe cancer pain receiving patient-controlled subcutaneous analgesia (PCSA). Mechanistically, we hypothesized that JK5G, by delivering inactivated bacteria and their bioactive metabolites, would beneficially reshape the gut ecosystem. This remodeling was anticipated to enhance the production of beneficial microbial metabolites with anti-inflammatory and immunomodulatory properties, thereby attenuating systemic inflammation and modulating pain pathways, ultimately leading to improved clinical outcomes. To comprehensively test this hypothesis and elucidate the underlying mechanisms, we employed a multi-omics approach. This included analyzing gut microbiota composition, fecal metabolomic profiles, and systemic immune markers. Furthermore, integrative analysis supplemented by machine learning techniques sought to identify potential biomarkers and provide a multidimensional understanding of the intervention’s impact on the gut-microbiome-immune axis. Through this design, our study seeks to provide robust clinical and mechanistic evidence for JK5G postbiotics as a novel, immunonutrition-based adjuvant therapy, addressing the pressing need for more effective and tolerable strategies in cancer pain management.

## 2 Methods

### 2.1 Patient enrollment

We conducted a randomized, double-blind, placebo-controlled trial at Chongqing University Cancer Hospital, China, from September 10, 2025, through November 10, 2025. The Ethics Committee of Chongqing University Cancer Hospital approved this study (CZLS2023084-A), and it is registered at http://www.chictr.org.cn/(ChiCTR2500108811). The study was prospectively registered, and the rapid enrollment reflects the high patient volume and efficient referral system of our center, which serves as a major regional cancer and cancer pain management hub. Among the 189 patients assessed for eligibility, 40 patients were excluded: 34 due to ineligibility and 6 due to unwillingness to participate (**Supplementary Figure 1**). Finally, 149 patients met all the inclusion criteria. These patients consented to participate and were then randomly and equally assigned to two treatment groups. One group (n = 75) received patient-controlled subcutaneous analgesia (PCSA) with hydromorphone plus placebo (Control Group), and the other group (n = 74) received PCSA plus JK5G postbiotics (JK5G Group).

Inclusion Criteria (1): Patients who have a confirmed diagnosis of cancer supported by clinical manifestations, including symptoms and signs, ancillary examinations such as serological markers including tumor markers, and pathological evaluations via tissue biopsy (2). Patients who are aged between 18 and 70 years and have been diagnosed with cancer pain by a certified pain specialist (3). Patients who have complete clinical records available (4). Patients who are conscious and able to cooperate (5). Patients who have cancer-related pain, as indicated by a Numerical Rating Scale (NRS) score of ≥4 recorded within the preceding 24 hours (6). Patients who are opioid-tolerant with an oral morphine-equivalent daily dosage of ≥60 mg and who are either experiencing inadequate pain relief or have intolerable side effects related to opioids (7). Patients who have an Eastern Cooperative Oncology Group (ECOG) Performance Status score ranging from 0 to 3 (8). Patients who can maintain adequate oral intake (9). Patients who have voluntarily provided informed consent and demonstrated adherence to the study protocol.

Exclusion Criteria (1): Patients who use or concurrently use nonsteroidal anti-inflammatory drugs (2). Patients who exhibit poor overall health status, such as an ECOG score greater than 3 (3). Patients who have received treatment with antibiotics, glucocorticoids, immunosuppressive drugs, or probiotics within the last three months (4). Patients who have undergone radiotherapy within the last month (5). Patients who present with gastrointestinal perforation, acute pancreatitis, acute intestinal obstruction, acute cerebral infarction, acute fever, or any acute or chronic infectious diseases (6). Patients who have liver or kidney insufficiency or congenital heart disease (7). Patients who have a documented history of psychiatric disorders (8). Pregnant or breastfeeding women (9). Patients who have a prior history of substance abuse or addiction (10). Patients who have known allergies to opioids (11). Patients who are unable to maintain adequate oral intake.

### 2.2 Study design and treatment

PCSA dosages and administration protocols were managed according to the NCCN clinical practice guidelines in adult cancer pain (6). Patients received 2.5 g of JK5G postbiotics or placebo once daily before meals for 7 days. Fecal samples were collected after 7 days of treatment. The JK5G microecological formulation is a postbiotic containing over 21 inactivated strains of Lactobacillus and their metabolic products (12). The JK5G preparation was acquired from JAPAN KYOWA INDUSTRIAL CO. LTD., located in Tokyo, Japan.

Both patients and investigators were blinded to the treatment administered in the study. The allocation sequence was produced by an interactive web response system, which also ensured that the sponsor remained unaware of the sequence. To maintain masking, both JK5G and the placebo were designed to be indistinguishable in appearance, a fact that was verified by an individual tasked with the allocation of investigational products. In cases of serious adverse events, emergency code breaking through the interactive web response system was allowed, or alternative therapies could be selected as subsequent lines of treatment.

To minimize confounding effects of diet and lifestyle on gut microbiota and metabolome profiles, all participants adhered to a standardized dietary and physical activity protocol for seven days prior to and during the study period. The dietary regimen was designed in strict accordance with the Nutrition Guidelines for Chinese Cancer Patients (2022) (13) and the Dietary Guidelines for Chinese Residents (2022) (14). To ensure uniformity and control, all meals for inpatients were centrally prepared and delivered by the hospital’s Department of Clinical Nutrition, with daily tray checks confirming a high meal completion rate (>90%). Physical activity levels were prescribed based on the ECOG Performance Status. This comprehensive protocol successfully standardized key background factors between the study groups. The complete standardized protocol, including detailed procedures for nutritional assessment, meal provision, activity prescription, and quantitative adherence monitoring, is provided in **Supplementary Material S1**.

Following enrollment in both groups, health-related QoL, nutritional status, pain levels, and psychological status were assessed before and after the treatment interventions. QoL was evaluated using the European Organization for Research and Treatment of Cancer Quality-of-Life Questionnaire Core 30 (EORTC QLQ-C30) version 3.0, which provides a comprehensive evaluation of various dimensions of QoL, including functional and symptomatic aspects post-treatment (3, 15). Nutritional status was assessed using the Patient-Generated Subjective Global Assessment (PG-SGA) (15). Pain levels were quantified using the NRS (3, 15). Psychological well-being over the preceding week was measured using the Hospital Anxiety and Depression Scale (HADS), which distinguishes between anxiety symptoms (HADS-A) and depressive symptoms (HADS-D), together with the Patient Health Questionnaire-9 (PHQ-9) (3, 15).

### 2.3 Trial outcomes

The primary outcomes were gut microbiota composition (assessed by 16S rRNA gene sequencing) and overall quality of life (QoL), measured using the validated EORTC QLQ−C30 questionnaire. The sample size was estimated based on the pain score of the QLQ−C30, as this domain is a sensitive and clinically meaningful indicator for detecting changes in cancer−pain interventions (16). Secondary outcomes included fecal metabolomic profiles, adverse events, serum inflammatory cytokines, and lymphocyte subsets.

### 2.4 Data collection

Comprehensive data regarding cytokine levels, lymphocyte subpopulations, complete blood counts, and serum biochemical parameters were systematically gathered from all participants involved in this study. Additionally, two independent researchers carefully reviewed the data collection forms to ensure the accuracy and reliability of the recorded information.

### 2.5 Sample size calculation

We determined that a sample of 135 subjects would provide 80% statistical power to detect an 8-point mean difference in the QLQ-C30 Pain score between groups with a two-sided significance level of 5% (12, 15). Subsequently, accounting for a potential 10% loss to follow-up, we enrolled an additional 14 participants, resulting in a total of 149 patients.

### 2.6 ELISA and flow cytometry

The serum concentrations of TNF-α, IL-2, IL-6, IL-8, and IL-10 were quantified using ELISA kits (R&D Systems, USA). Common tumor markers (CEA, CYFRA 21-1, CA125, CA19-9, and NSE) were measured in serum samples using a chemiluminescence immunoassay on a fully automated clinical analyzer according to the manufacturer’s instructions. Total protein, prealbumin, and albumin levels were assessed separately using appropriate assays. The analysis of T lymphocyte subsets was conducted using ten-color flow cytometry (BD FACS Canto II), employing a lyse/no-wash protocol, as previously outlined (12). Each measurement, including cytokine quantification and flow cytometry analysis, was conducted a minimum of three times to ensure consistency and reliability.

### 2.7 Fecal sample DNA extraction, PCR amplification and 16S rRNA-seq

All fecal specimens were preserved at −80 °C prior to DNA extraction and subsequent analysis. Microbial DNA was extracted from the fecal samples using the E.Z.N.A.^®^ Soil DNA Kit in accordance with the manufacturer’s guidelines. The concentration and purity of the extracted DNA were assessed using a NanoDrop 2000 UV–vis spectrophotometer, and DNA quality was evaluated through 1% agarose gel electrophoresis. The V3–V4 hypervariable regions of the bacterial 16S rRNA gene were amplified using specific primers on a thermocycler PCR system, with each PCR reaction carried out in triplicate using a defined reaction mixture following a standardized protocol. The resultant PCR products were extracted from the agarose gel, purified, and then quantified. Purified amplicons were sequenced on the Illumina PE300 platform (Illumina, USA) using paired-end sequencing technology. Data processing, amplicon sequence variants (ASVs) clustering, and taxonomic analysis were performed sequentially using the MG Sunshine Cloud Analysis Platform. Subsequently, various diversity indices assessments, including Principal Coordinate Analysis (PCoA), Linear Discriminant Analysis Effect Size (LEfSe), and redundancy analyses (RDA), were conducted using QIIME and R software (17, 18). Through the application of the LEfSe test, microbial groups were differentiated based on their distinctive microbial features. The relationship between bacterial community structures and environmental variables was explored using the RDA approach (17).

### 2.8 Sample preparation for untargeted metabolomics analysis

Fecal samples, previously stored at –80 °C, were removed and allowed to thaw at ambient temperature. The subsequent procedures were carried out by Majorbio Bio-Pharm Technology Co., Ltd. A total of 50 mg of each fecal sample was used for the analysis. To achieve homogenization, 400 μL of a methanol-water mixture (4:1, v/v) was added, and the sample was processed with a homogenizer for 10 seconds. After ultrasonic extraction of the mixture on ice for 10 minutes, the mixture was stored at –20 °C for 30 minutes before centrifugation. The sample was then centrifuged for 15 minutes, at 13,000 rpm at 4 °C, and 200 μL of the resulting supernatant was reserved for liquid chromatography-mass spectrometry (LC-MS) analysis. A Quality Control (QC) sample was generated by combining aliquots from all the samples to create a pooled specimen. This pooled QC sample was subsequently analyzed using the same methodology as the analytical samples. QC samples were injected at regular intervals (after every 10 analytical samples) throughout the analytical run to establish a dataset that facilitates the assessment of repeatability.

### 2.9 LC-MS analysis parameters for untargeted metabolomics

The subsequent stages were also managed by Majorbio Bio-Pharm Technology Co., Ltd. The LC-MS analysis was conducted using the AB Sciex TripleTOF 5600TM mass spectrometer system (AB SCIEX). An Acquity BEH C18 column (100 mm × 2.1 mm, i.d., 1.7 µm; Waters) was used and maintained at a temperature of 40 °C. Separation was achieved through a gradient elution, progressing from 5% solvent B to 20% solvent B over the first 3 minutes, then increasing from 20% B to 95% B over the next 6 minutes. The gradient was maintained at 95% B for 4 minutes. It was then transitioned back to 5% B over 0.1 minutes and maintained at this level for a total duration of 2.9 minutes at a flow rate of 0.40 mL/min. Solvent B consisted of acetonitrile and isopropanol in a 1:1 ratio supplemented with 0.1% (v/v) formic acid, while solvent A consisted of an aqueous solution of formic acid at the same concentration. An injection volume of 20 μL was employed. Mass spectrometry data acquisition was performed using the AB Sciex TripleTOF 5600TM system equipped with an electrospray ionization (ESI) source operating in both positive and negative ionization modes. The capillary voltages were set at 5000 V and –4000 V, respectively. The cone voltage was set at 40 V with a collision energy of 5 eV. The source temperature was adjusted to 500 °C with a desolvation gas flow rate of 45 L/h. Centroid data were collected across a mass-to-charge ratio (m/z) range of 50 to 1000 with a resolution of 30,000. Prior to pattern recognition analyses, the raw data underwent preprocessing steps including baseline filtering, peak identification, integration, retention time correction, peak alignment, and normalization. These steps were performed using the Progenesis QI metabolomics processing software (Waters). After preprocessing, the positive and negative datasets were analyzed using the SIMCA-P software suite, with metabolites compared via orthogonal partial least squares discriminant analysis (OPLS-DA).

### 2.10 Metabolite set enrichment analysis

Metabolite Set Enrichment Analysis (MSEA) was performed using the MetaboAnalyst R package (19). Through the Over Representation Analysis (ORA) mode of MSEA and based on the Small Molecule Pathway Database (SMPDB), all identified metabolites from the comparison groups were analyzed to help determine and interpret changing patterns of metabolite concentrations within significant biological pathways. This approach enabled the identification of metabolomic pathways that were significantly enriched.

### 2.11 Model-based integration of metabolite observations and species abundances 2

Model-based Integration of Metabolite Observations and Species Abundances 2 (MIMOSA2), introduced by the Borenstein Lab, is a computational framework that leverages microbiome data and reference databases to construct metabolic models (20). The method first calculates the community-wide metabolite potential (CMP), representing the collective metabolic capacity of microbial species to produce or utilize specific metabolites. By building a predictive metabolic model, MIMOSA2 estimates how microbial community composition influences metabolite concentrations and evaluates the consistency between predicted and experimentally measured metabolomic profiles, thereby establishing a functional link between species abundance and metabolite variation. In this study, the MIMOSA2 R package was employed to analyze the multi-omics data.

### 2.12 Model building based on machine-learning

Potential biomarkers exhibiting high sensitivity, accuracy, and stability were identified using the optimal machine learning model. Four commonly used algorithms—support vector machine (SVM), random forest (RF), logistic regression (LR), and Least Absolute Shrinkage and Selection Operator (LASSO) were implemented with ten-fold cross-validation in R to construct models based on genus or metabolite abundance data (21). During model construction, the microbiome or metabolome dataset was initially partitioned into ten subsets. In each cross-validation iteration, seven subsets were randomly selected as the training set for model development, while the remaining three subsets served as an independent test set for validation. Model performance was evaluated using receiver operating characteristic (ROC) curves and the area under the curve (AUC). Subsequently, the overlapping biomarkers selected by multiple algorithms were identified using Venn analysis to enhance the robustness of the selection process. The construction of data preprocessing and model prediction was performed in R software. Subsequently, features were ranked by importance scores, and subsets with varying numbers of top-ranked features were used for modeling to determine the most parsimonious model configuration.

### 2.13 SHAP analysis

SHapley Additive exPlanations (SHAP) is a technique utilized for elucidating the prediction outcomes of machine learning models, aiming to determine, for each prediction rendered by the model, the extent of contribution from each input feature to that prediction result (the SHAP value) (22). The research employed a repeated ten-fold cross-validation approach, partitioning the training dataset into ten equally sized segments. In each cycle of cross-validation, seven segments were designated as the training set, whereas the remaining three segments functioned as the validation set to evaluate model performance. This value offers a lucid indication of which features are paramount for a specific prediction result and whether their influence is positive or negative. The principal advantage of the SHAP method lies in its capacity to furnish both local interpretability for individual prediction results and global interpretability concerning the overall decision-making framework of the model.

### 2.14 Statistical analysis

Data analysis was performed using SPSS version 24.0 and R version 4.3.3. Descriptive statistics were employed to calculate frequencies, means, and standard deviations. For categorical variables, differences between groups regarding baseline characteristics and clinical outcomes were evaluated using Fisher’s exact test and χ² tests. Continuous variables were assessed through independent-sample Student’s t-tests. A p-value of less than 0.05 was deemed statistically significant, with the following designations: *p < 0.05, **p < 0.01, ***p < 0.001, and ns indicates no statistically significant difference.

Metabolite mass spectra were identified by leveraging accurate mass, MS/MS fragment spectra, and isotope ratio discrepancies. Searches were conducted in reputable biochemical databases such as the Human Metabolome Database (HMDB, http://www.hmdb.ca/) and the Metlin database (https://metlin.scripps.edu/). The selection of metabolites exhibiting significant differences was based on the Variable Importance in Projection (VIP) score derived from the Partial Least Squares Discriminant Analysis (PLS-DA) model and the *p*-value from the Student’s t-test. Metabolites with a VIP score exceeding 1 and a p-value of less than 0.05 were classified as significantly-different metabolites. The biological pathways associated with these differential metabolites were identified by functional annotation using the KEGG database (https://www.kegg.jp/kegg/pathway.html). Pathway enrichment analysis was conducted using the Python software package scipy.stats (https://docs.scipy.org/doc/scipy/). Fisher’s exact test was utilized to identify the biological pathways most relevant to the experimental treatment.

## 3 Results

### 3.1 Baseline characteristics of patients

A total of 149 patients were enrolled, received the allocated treatments, and were included in all efficacy and safety analyses. The cohort comprised 75 patients receiving PCSA plus placebo (control group) and 74 patients receiving PCSA plus JK5G postbiotics (JK5G group). The baseline demographic and clinical characteristics were well-balanced between the two groups, with no statistically significant differences in age, sex, BMI, ECOG performance status, smoking history, primary cancer type, or cancer stage. Regarding the distribution of primary tumor types, lung cancer was the most common in both the control (n=62) and JK5G (n=59) groups. Other malignancies included colorectal cancer (control: 3, JK5G: 5), gastric cancer (2 vs. 2), pancreatic cancer (1 vs. 3), and other tumor types (7 vs. 5). Given that lung cancer constituted the predominant subgroup, a subsequent subgroup analysis focusing on these patients was performed. Critically, there were no significant differences between the groups in baseline pain scores (**Table 1**).

**Table 1:** Patients’ demographic and baseline characteristics.

Characteristic	Control group (n=75)	JK5G group (n=74)	*t/*χ^2^*/Z*	*P*
Age, years	63.85 ± 10.00	65.58 ± 9.47	-1.08	0.281
Sex—no.(%)			1.83	0.176
Male	59 (78.67%)	51 (68.92%)		
Female	16 (21.33%)	23 (31.08%)		
Height,cm	162.39 ± 7.22	160.37 ± 7.63	1.65	0.101
Weight, kg	55.79 ± 8.85	55.55 ± 9.77	0.16	0.872
BMI, kg/m 2	21.13 ± 2.87	21.62 ± 3.68	-0.92	0.362
ECOG—no.(%)			3.76	0.153
1	42 (56.00%)	31 (41.90%)		
2	24 (32.00%)	35 (47.30%)		
3	9 (12.00%)	8 (10.81%)		
Smoking status—no. (%)			2.96	0.085
Former	29 (38.67%)	39 (52.70%)		
Never	46 (61.33%)	35 (47.30%)		
Primary cancer—no.(%)			1.90	0.754
Lung	62 (82.67%)	59 (79.73%)		
Colorectal	3 (4.00%)	5 (6.76%)		
Gastric	2 (2.67%)	2 (2.70%)		
Pancreatic	1 (1.33%)	3 (4.05%)		
Other	7 (9.33%)	5 (6.76%)		
AJCC cancer stage—no. (%)			4.36	0.113
IIIB	1 (1.33%)	2 (2.70%)		
IIIC	4 (5.33%)	0		
IV	70 (93.33%)	72 (97.30%)		
NRS score—no. (%)			0.06	0.813
No pain (0)	0	0		
Mild pain (1-3)	0	0		
Moderate pain (4–6)	57 (76.00%)	55 (74.32%)		
Severe pain (7-10)	18 (24.00%)	19 (25.68%)		

Data are means ± SD or n (%). Percentages might not total 100% because of rounding. ECOG, Eastern Cooperative Oncology Group; AJCC, American Joint Committee on Cancer; NRS, Numerical Rating Scale.

### 3.2 Vital characteristics of patients after treatment

After treatment, nutrition was assessed using the PG-SGA, and pain intensity was measured by the NRS. The pain intensity score significantly improved in the JK5G group compared to the control group (*p* = 0.014, **Table 2**). Although there was no statistically significant difference in the overall PG-SGA grade distribution between groups (*p* = 0.056), a descriptive analysis revealed a trend toward improvement in the JK5G group. The distribution of patients across PG-SGA categories was as follows: scores ≥9 (control: 61, JK5G: 61), 4–8 (10 vs. 3), 2–3 (4 vs. 7), and 0–1 (0 vs. 3). Mood symptoms, assessed by the HADS-D and the PHQ-9 depression scores, also showed no significant differences (**Table 2**). However, JK5G postbiotics treatment significantly improved QoL compared to the control group. Specifically, patients receiving JK5G showed better cognitive functioning (90.62 ± 13.84 vs. 85.23 ± 14.24, *p* = 0.023), improved social functioning (55.92 ± 33.68 vs. 41.69 ± 30.98, *p* = 0.010), lower pain intensity scores (43.79 ± 30.54 vs. 56.37 ± 24.04, *p* = 0.007), and fewer financial difficulties (45.15 ± 32.20 vs. 57.56 ± 32.19, *p* = 0.023) than the control group (**Table 2**). These results indicate that JK5G postbiotics treatment may improve QoL in patients with moderate to severe cancer pain.

**Table 2:** Analyses of patients’characteristics after treatment.

Characteristic	Control group (n=75)	JK5G group (n=74)	*t/*χ^2^*/Z*	*P*
PG-SGA score—no. (%)			7.58	0.056
0–1	0	3 (4.05%)		
2–3	4 (5.33%)	7 (9.50%)		
4–8	10 (13.33%)	3 (4.05%)		
≥9	61 (81.33%)	61 (82.43%)		
NRS score—no. (%)			6.07	0.014
No pain (0)	0	0		
Mild pain (1-3)	39 (52.00%)	53 (71.62%)		
Moderate pain (4–6)	36 (48.00%)	21 (28.38%)		
Severe pain (7-10)	0	0		
Assessment of mood symptoms				
HADS				
Anxiety subscale (HADS-A)	3.23 ± 3.05	2.35 ± 2.96	1.65	0.101
Depression subscale (HADS-D)	4.55 ± 3.56	3.73 ± 2.99	1.41	0.162
PHQ-9 Depression severity			5.11	0.076
No (0-4)	60 (80.00%)	63 (85.14%)		
Mild (5-9)	10 (13.33%)	11 (14.87%)		
Moderate (10-14)	5 (6.66%)	0		
EORCT QLQ-C30				
Functional Scales				
Physical Functioning	65.27 ± 23.00	71.78 ± 25.27	-1.61	0.110
Role Functioning	44.10 ± 31.94	54.34 ± 36.26	-1.79	0.076
Emotional Functioning	93.44 ± 8.91	95.34 ± 7.38	-1.39	0.166
Cognitive Functioning	85.23 ± 14.24	90.62 ± 13.84	-2.29	0.023
Social Functioning	41.69 ± 30.98	55.92 ± 33.68	-2.63	0.010
Global Health	63.01 ± 11.54	62.63 ± 15.93	0.17	0.870
Symptom Scales				
Fatigue	22.67 ± 16.10	18.73 ± 17.17	1.42	0.195
Nausea and Vomiting	7.16 ± 18.09	5.26 ± 16.41	0.66	0.512
Pain	56.37 ± 24.04	43.79 ± 30.54	2.73	0.007
Dyspnoea	14.19 ± 19.23	12.25 ± 18.78	0.61	0.543
Insomnia	22.30 ± 23.92	17.73 ± 23.61	1.15	0.252
Appetite Loss	36.14 ± 30.52	30.96 ± 33.51	0.97	0.336
Constipation	22.23 ± 20.20	20.86 ± 23.19	0.38	0.708
Diarrhoea	0.47 ± 3.94	2.76 ± 18.81	-1.67	0.097
Financial Difficulties	57.56 ± 32.19	45.15 ± 32.20	2.30	0.023

Data are means ± SD or n (%). Percentages might not total 100% because of rounding. Abbreviations: PG-SGA, Patient-Generated Subjective Global Assessment; NRS, Numerical Rating Scale; HADS, Hospital Anxiety and Depression Scale; PHQ-9, Patient Health Questionnaire-9; EORCT QLQ-C30, European Organization for Reasearch and Treatment of Cancer Quality-of-Life Questionnaire Core 30.

### 3.3 Safety

Adverse events (AEs) observed in both groups were similar in terms of type, severity, and frequency. These AEs were generally well tolerated by patients and did not significantly impact treatment adherence. Furthermore, no unforeseen adverse events were documented, and the incidence of opioid-related adverse events was not significantly different between groups, as shown in **Table 3**. Additionally, there were no instances of suicide attempts or opioid misuse among patients treated with PCSA.

**Table 3:** Frequency of main adverse effects related to opioids.

Adverse events	Control group (n=75)	JK5G group (n=74)	χ^2^	*P*
Total events	15 (20.00%)	13 (17.57%)	0.14	0.704
Constipation	13 (17.33%)	12 (16.22%)	0.03	0.855
Nausea	1 (1.33%)	1 (1.35%)	<0.01	0.992
Vomiting	1 (1.33%)	0	0.99	0.320

Data are presented in n (%).

### 3.4 JK5G postbiotics modified gut microbial communities and functional prediction in patients with moderate to severe cancer pain

**Figure 1:**
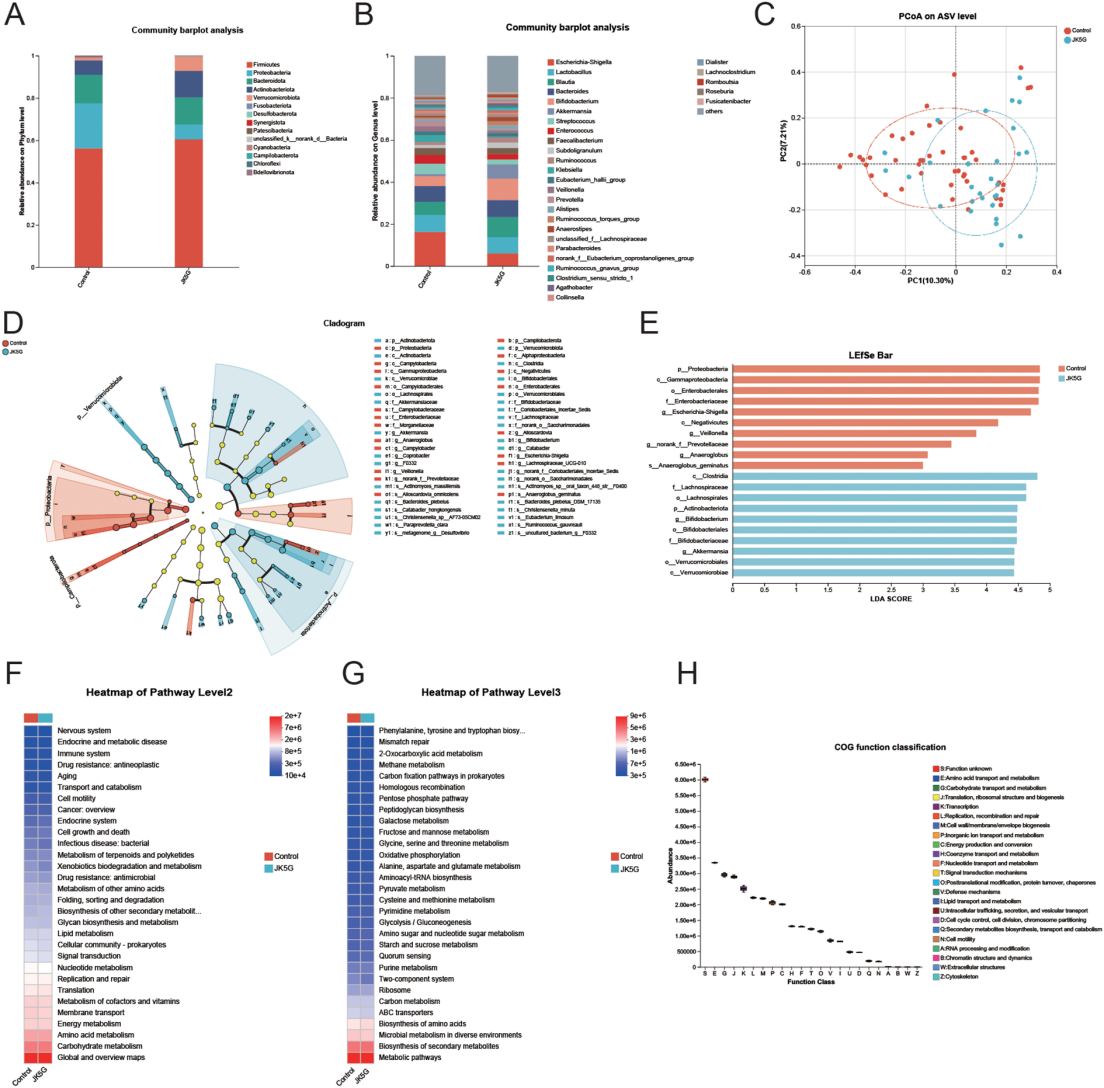
Effects of JK5G postbiotics on gut microbiota composition and function in cancer pain patients. **(A)** Phylum-level and **(B)** genus-level taxonomic distribution. **(C)** Principal coordinate analysis (PCoA) based on unweighted UniFrac distance (ASV level). **(D)** Cladogram of gut microbiota (JK5G vs control groups). **(E)** Linear Discriminant Analysis (LDA) scores from LEfSe showing biomarker taxa (LDA score > 2, *p* < 0.05 by Wilcoxon signed-rank test). **(F)** KEGG pathway predictions (Level 2) and **(G)** Level 3 using PICRUSt2. **(H)** COG function predictions using PICRUSt2.

We analyzed 4,983,306 sequences, and the rarefaction diversity results showed that most of the microbial diversity was captured (**Supplementary Figure 2A**). No significant difference was observed in taxonomic alpha diversity, including Chao1 indices, Abundance-based Coverage Estimator (ACE), and Shannon indices, during the research period (**Supplementary Figures 2B–D**). Subsequent analysis of the intestinal microflora profile revealed that the composition (rather than the overall diversity) of the bacterial community changed substantially among different samples (**Figures 1A, B**). Principal Coordinates Analysis (PCoA), a key component of β-diversity analysis, showed that the gut microbiota composition in the JK5G treatment group did not differ significantly across study periods (**Figure 1C**). Additionally, the LEfSe test and cladogram plots showed that the pathogenic bacteria *Escherichia-Shigella* and *Veillonella* increased in the control group, whereas short-chain fatty-acid (SCFA)-producing bacteria, including *Bifidobacterium* and the beneficial commensal *Akkermansia muciniphila*, increased in the JK5G group after treatment (**Figures 1D, E**).

Based on the 16S amplicon sequencing data combined with the KEGG and COG databases, we predicted the biological functions of the bacteria (**Figures 1F, H**). The 16S rRNA sequencing data combined with KEGG functional predictions indicated that bacterial colony function is primarily related to the pathway of “Carbohydrate metabolism”, “Amino acid metabolism” and “Energy metabolism” (**Figure 1F**). Combined with information from KEGG data at a deeper level (3rd Level), we could further confirm the association of bacteria with the “Biosynthesis of amino acids”, “Biosynthesis of secondary metabolites”, and “Microbial metabolism in diverse environments” (**Figure 1G**). In combination with the COG database, the functions of bacteria were predicted to be mainly involved in metabolism, with amino acid metabolism accounting for the highest abundance (**Figure 1H**). These data suggest that JK5G postbiotics could restructure the gut flora composition and function of patients with moderate to severe cancer pain by enhancing beneficial commensals while suppressing pathogenic species, which may have indirect implications for the tumor microenvironment via systemic immune and metabolic modulation.

### 3.5 Identification of microbiome biomarkers via machine learning approaches

**Figure 2:**
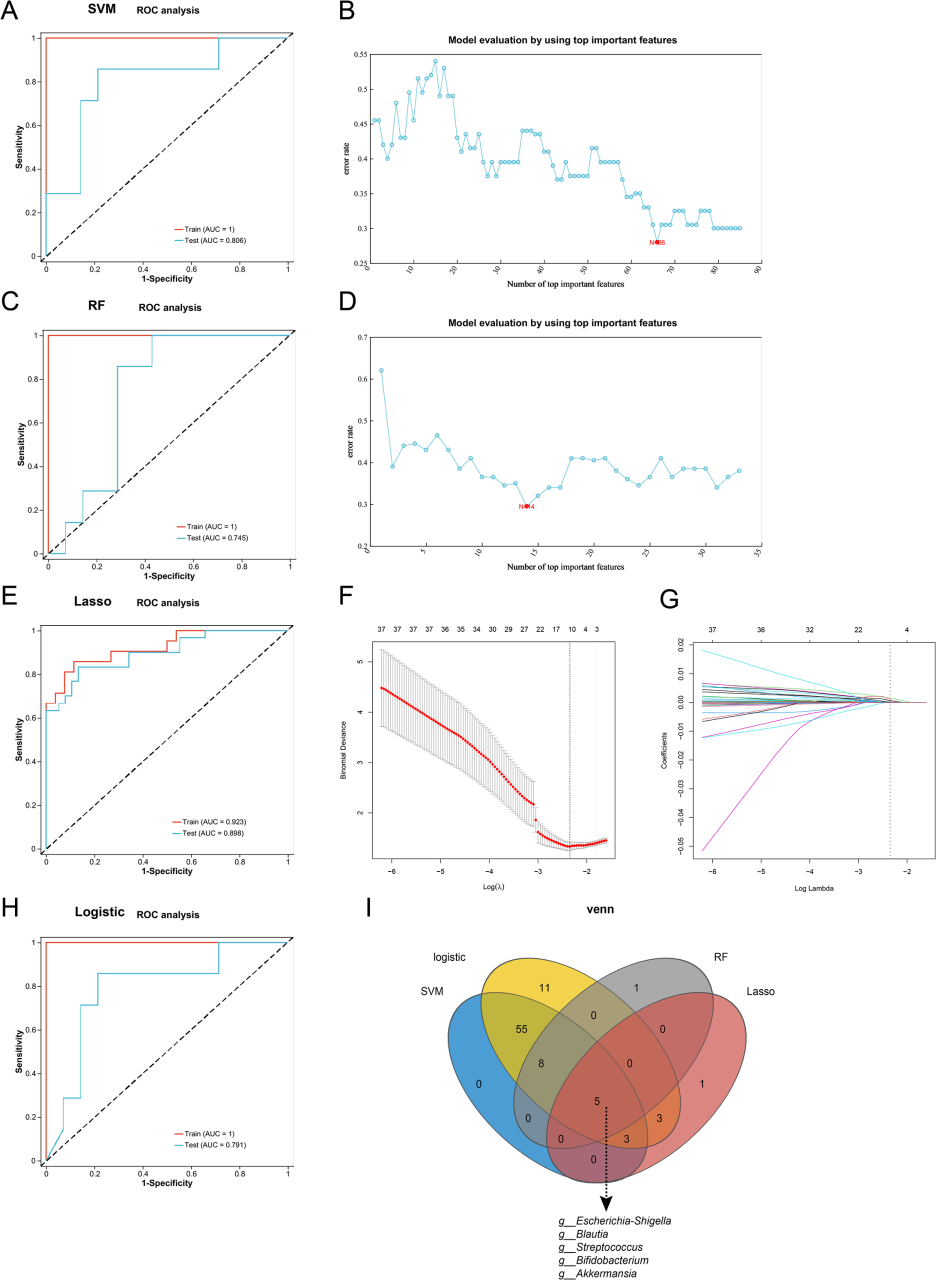
Machine learning model development and validation for microbiome biomarker identification. Four machine learning algorithms were used to identify unique biomarkers: **(A, B)** Support Vector Machine (SVM), **(C, D)** Random Forest (RF), **(E–G)** LASSO, and **(H)** Logistic Regression (LR). **(I)** Venn analysis was applied to select common biomarker variables and create a common set. ROC curves (left) show model performance in training and testing validation, with AUC values indicating predictive accuracy (higher AUC represents better performance). Top important features were used to evaluate the SVM **(B)** and RF **(D)** models. Variable trajectories **(F)** and prognostic risk plots **(G)** are derived from the LASSO regression model.

The above results have demonstrated that the relative abundances of microorganisms in JK5G group changed significantly. These differential microorganisms were then used to build classification models through four machine learning methods (SVM, RF, LASSO, and logistic regression), and the ROC curve and the AUC were used to evaluate the model performance. Bar chart evaluation demonstrated that all four machine learning models achieved satisfactory performance in microbiome biomarker selection (**Supplementary Figure 3**). The four machine learning models demonstrated robust performance on both the training and test sets. On the training set, the AUC values were 1.0, 1.0, 0.923, and 1.0 for SVM, RF, LASSO, and logistic regression, respectively (**Figures 2A, C, E, H**). Correspondingly, on the test set, the AUC values were 0.806, 0.745, 0.898, and 0.791. Based on the feature selection results from the four machine learning algorithms, SVM identified 66 microbial species (**Figure 2B**), RF selected 14 (**Figure 2D**), LASSO identified 11 (**Figures 2F, G**), and Logistic Regression selected 85 species. The intersection of features selected by all four methods, determined via Venn analysis, revealed five overlapping species: *Akkermansia muciniphila*, *Bifidobacterium, Escherichia-Shigella*, *Blautia*, and *Streptococcus*, suggesting their potential as biomarkers (**Figure 2I**). Notably, *Akkermansia muciniphila*, *Bifidobacterium*, and *Escherichia-Shigella* were particularly prominent in the differential abundance analysis between the JK5G and control groups (**Figure 1E**). These findings indicate that these five microbial species may serve as reliable exploratory biomarkers for further investigation.

### 3.6 JK5G postbiotics modulate immune responses and inflammatory cytokines, with SHAP analysis identifying *Akkermansia muciniphila* and *Bifidobacterium* as key exploratory biomarkers

**Figure 3:**
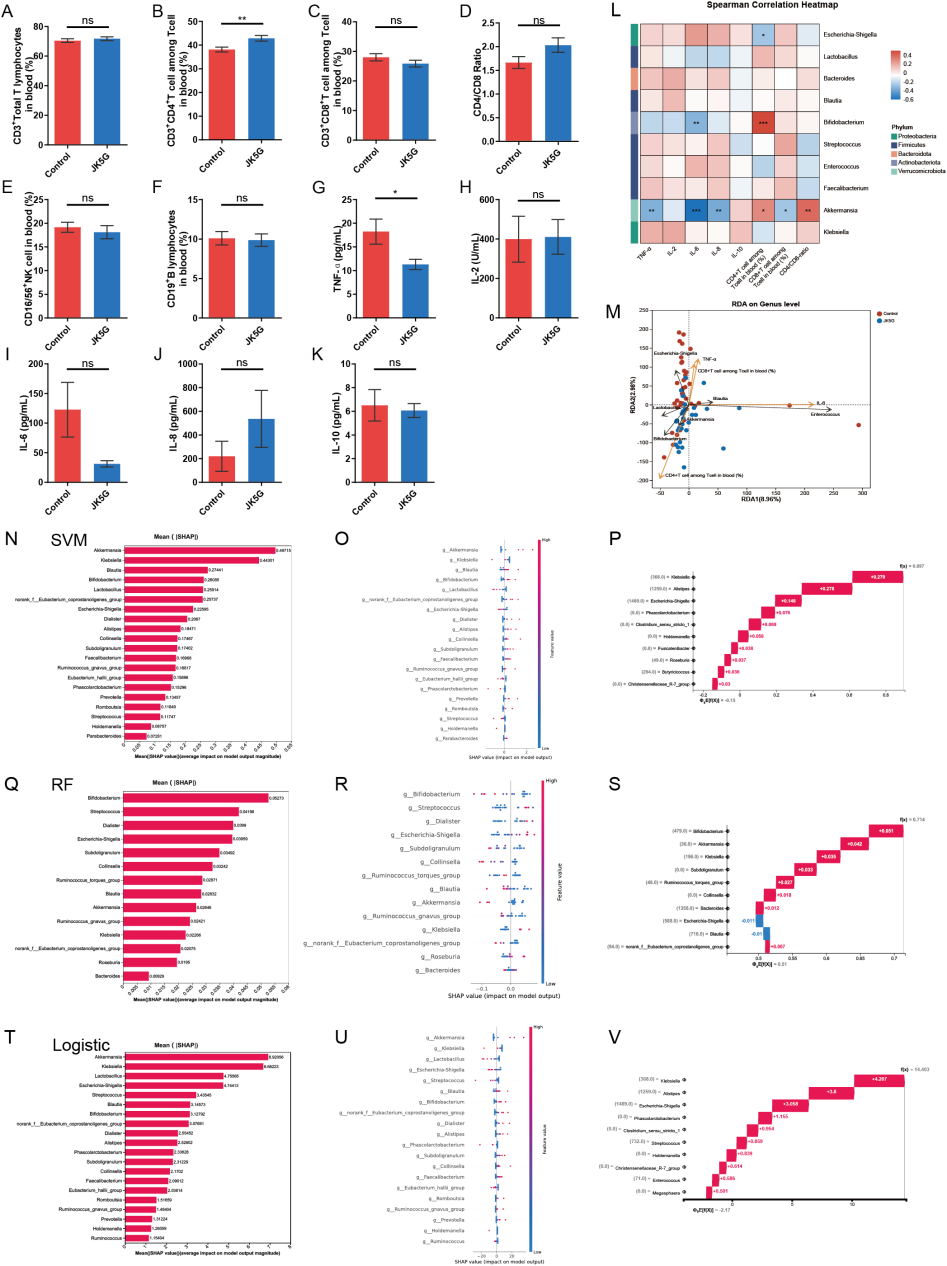
JK5G Postbiotics Modulate Immune Parameters in Cancer Pain Patients: Insights from Interpretable Machine Learning (SHAP Analysis of SVM, RF, and Logistic Regression Models). **(A–F)** Proportions of CD3^+^ T cells **(A)**, CD3^+^CD4^+^ T cells **(B)**, CD3^+^CD8^+^ T cells **(C)**, CD4/CD ratio **(D)**, CD16/56^+^ NK cells **(E)**, and CD19^+^ B cells **(F)** in peripheral blood (JK5G vs. control). **(G–K)** Serum levels of TNF-α **(G)**, IL-2 **(H)**, IL-6 **(I)**, IL-8 **(J)**, and IL-10 **(K)** (JK5G vs. control). **(L)** Correlation heatmap between immune cells, cytokines, and bacterial genera (red: positive, blue: negative, white: no correlation). **(M)** Redundancy analysis (RDA) of samples, microbial species, and physiological factors (black arrows: bacteria; symbols: samples; yellow arrows: factors; axes show variance percentages). SHAP analysis was applied to three models: SVM **(N–P)**, RF **(Q–S)**, and Logistic Regression **(T–V)**. Panels display global feature importance (left), summary plots (middle), and local explanations (right) for each model. Global importance (left) is based on mean absolute SHAP values, summarizing average feature impacts. Summary plots (middle) show feature effect directions and heterogeneous influences. Waterfall plots (right) illustrate individual prediction deviations from the base value, demonstrating local additivity of feature contributions. Data **(A–K)** are means ± SEMs; *p < 0.05, **p < 0.01, ***p < 0.001, ns, not significant.

Emerging evidence has shown that inflammatory cytokines and the function of T cell subsets play a fundamental role in mediating cancer-related pain (9). To investigate the immunomodulatory effects of the postbiotic JK5G, we performed a comprehensive analysis of peripheral blood immune cells (**Figures 3A–F**), serum inflammatory cytokines (**Figure 3G–K**), and nutritional biomarkers (**Supplementary Figures 5**) in patients with moderate-to-severe cancer-related pain. Flow cytometry analysis revealed that JK5G administration significantly increased the percentage of CD3^+^CD4^+^ T cells (**Figure 3B**, p < 0.01) and decreased serum levels of the pro-inflammatory cytokine TNF-α (**Figure 3G**) compared to the control group. No significant differences were observed in other immune cell subsets (CD3^+^ total T lymphocytes, CD3^+^CD8^+^ T cells, CD16/56^+^ NK cells, CD19^+^ B lymphocytes; **Figures 3A, C–F**) or in serum nutritional markers (total protein, albumin, prealbumin; **Supplementary Figures 4A–C**), or in common tumor markers (CEA, CYFRA 21-1, CA125, CA199, NSE; **Supplementary Figures 5A–C**).

Correlation analysis indicated that these immunomodulatory effects were associated with specific changes in the gut microbiota (**Figure 3L**). *Akkermansia muciniphila* abundance was positively correlated with the percentage of CD3^+^CD4^+^ T cells and the CD4/CD8 ratio, and negatively correlated with serum levels of TNF-α, IL-6, IL-8 and the percentage of CD3^+^CD8^+^ T cells. *Bifidobacterium* showed a positive correlation with CD3^+^CD4^+^ T cells and a negative correlation with IL-6. In contrast, *Escherichia-Shigella* was negatively correlated with CD3^+^CD4^+^ T cells.

To further visualize and validate these multivariate relationships, a Redundancy Analysis (RDA) was conducted (**Figure 3M**). The RDA plot confirmed the significant correlations: *Akkermansia muciniphila* and *Bifidobacterium* exhibited positive associations with the percentage of CD3^+^CD4^+^ T cells and negative associations with serum TNF-α, whereas *Escherichia-Shigella* showed an opposite correlation pattern.

To definitively identify the gut microbial taxa most critically predictive of the JK5G intervention, we employed interpretable machine learning using SHAP analysis across multiple models. Summary plots of global feature importance (**Figures 3N, Q, T**) revealed that *Akkermansia muciniphila* was the strongest predictor in both the SVM (SHAP value: +0.49715) and Logistic Regression (SHAP value: +6.92056) models. In contrast, *Bifidobacterium* exhibited the highest contribution in the RF model (SHAP value: +0.05273). The top five predictive features for each model were enumerated: for SVM—*Akkermansia muciniphila*, *Klebsiella*, *Blautia*, *Bifidobacterium*, *Lactobacillus* (**Figure 3N**); for RF—*Bifidobacterium*, *Streptococcus*, *Dialister*, *Escherichia-Shigella*, *Subdoligranulum* (**Figure 3Q**); for Logistic Regression—*Akkermansia muciniphila*, *Klebsiella*, *Lactobacillus*, *Escherichia-Shigella*, *Streptococcus* (**Figure 3T**).

SHAP summary plots (**Figures 3O, R, U**) visualized the directionality and distribution of each feature’s contribution, confirming *Akkermansia muciniphila* as the most important feature in the SVM and Logistic Regression models and *Bifidobacterium* as the top contributor in the RF model. Furthermore, analysis of predictive performance (**Figures 3P, S, V**) indicated that model outputs were predominantly driven by a core set of key features, with *Klebsiella* being the most influential single factor in the SVM and Logistic Regression models, while *Bifidobacterium* and *Akkermansia muciniphila* were paramount in the RF model.

Collectively, these results demonstrate that JK5G postbiotic treatment alleviates systemic inflammation and enhances CD4^+^ T cell levels. These changes are associated with a restructuring of the gut microbiota, as evidenced by correlation and RDA analyses. Critically, interpretable machine learning (SHAP) convergently identifies *Akkermansia muciniphila* and *Bifidobacterium* as the key exploratory microbial biomarkers for the intervention’s effect, providing a robust, data-driven link between specific microbial modulation and the observed immune improvement.

### 3.7 Subgroup analysis of patients with lung cancer

Given that lung cancer was the predominant subgroup in our cohort, we performed a dedicated analysis to evaluate the efficacy and safety of JK5G postbiotics in these patients. The baseline characteristics of the lung cancer subgroup (Control: n=62, JK5G: n=59) were well-matched, with no significant differences in age, sex, BMI, ECOG performance status, cancer stage, or baseline pain scores (**Table 4**). Additionally, there were no statistically significant differences in smoking history, histology, or anticancer therapy between the two groups at baseline. Regarding histology, the distribution of adenocarcinoma, squamous cell carcinoma, and other types was 41 vs. 38, 8 vs. 12, and 13 vs. 9 in the control and JK5G groups, respectively. In terms of anticancer therapy received during the intervention period, the numbers of patients undergoing chemotherapy and targeted therapy were 38 vs. 36 and 23 vs. 23 in the control and JK5G groups, respectively.

**Table 4:** Demographic and baseline characteristics of subgroup patients with Lung cancer.

Characteristic	Control group (n=62)	JK5G group (n=59)	*t/*χ^2^*/Z*	*P*
Age, years	63.77 ± 10.39	65.36 ± 9.58	-0.87	0.386
Sex—no. (%)			0.62	0.432
Male	48 (77.42%)	42 (71.19%)		
Female	14 (22.58%)	17 (28.81%)		
Height,cm	161.95 ± 7.26	160.74 ± 7.11	0.92	0.359
Weight, kg	55.95 ± 8.94	56.25 ± 9.61	-0.18	0.861
BMI, kg/m 2	21.29 ± 2.89	21.82 ± 3.80	-0.86	0.391
ECOG—no. (%)			5.20	0.074
1	36 (56.00%)	25 (41.90%)		
2	19 (32.00%)	30 (47.30%)		
3	7 (12.00%)	4 (10.81%)		
Smoking status—no.(%)			2.91	0.088
Former	21 (33.87%)	29 (49.15%)		
Never	41 (66.13%)	30 (50.85%)		
Histology—no. (%)			1.57	0.457
Adenocarcinoma	41 (66.13%)	38 (64.41%)		
Squamous cell	8 (12.00%)	12 (20.34%)		
Other	13 (20.97%)	9 (15.25%)		
AJCC cancer stage—no. (%)			4.08	0.130
IIIB	1 (1.61%)	1 (1.69%)		
IIIC	3 (4.84%)	0		
IV	58 (93.55%)	58 (98.31%)		
Anticancer therapy—no. (%)			0.05	0.831
Chemotherapy	39 (62.90%)	36 (61.02%)		
Targeted therapy	23 (37.10%)	23 (38.98%)		
NRS score—no. (%)			0.01	0.942
No pain (0)	0	0		
Mild pain (1-3)	0	0		
Moderate pain (4–6)	48 (77.42%)	46 (77.97%)		
Severe pain (7-10)	14 (22.58%)	13 (22.03%)		

Data are means ± SD or n (%). Percentages might not total 100% because of rounding. Abbreviations: ECOG, Eastern Cooperative Oncology Group; AJCC, American Joint Committee on Cancer; NRS, Numerical Rating Scale.

The beneficial effects of JK5G postbiotics observed in the overall cohort were not only preserved but appeared more pronounced in the lung cancer subgroup. Patients receiving JK5G demonstrated a significant reduction in pain intensity scores compared to the control group (*p* < 0.05, **Table 5**). Furthermore, JK5G treatment was associated with significant improvements in several QoL domains. Notably, in addition to the improvements in cognitive functioning, social functioning, pain intensity, and financial difficulties observed in the overall analysis, role functioning also showed a statistically significant improvement in the lung cancer subgroup (all *p* < 0.05, **Table 5**). These results indicate that the primary benefits of JK5G on pain relief and QoL enhancement are robust and potentially even more significant within this major patient subgroup.

**Table 5:** Characteristics of patients with lung cancer after treatment: subgroup analysis.

Characteristic	Control group (n=62)	JK5G group (n=59)	*t/*χ^2^*/Z*	*P*
PG-SGA score—no. (%)			7.56	0.056
0–1	0	3 (5.08%)		
2–3	4 (6.45%)	7 (11.86%)		
4–8	10 (16.13%)	3 (5.08%)		
≥9	48 (77.42%)	46 (77.97%)		
NRS score—no. (%)			8.73	0.003
No pain (0)	0	0		
Mild pain (1-3)	30 (48.39%)	44 (74.58%)		
Moderate pain (4–6)	32 (51.61%)	15 (25.42%)		
Severe pain (7-10)	0	0		
Assessment of mood symptoms				
HADS				
Anxiety subscale (HADS-A)	2.16 ± 2.78	3.02 ± 2.99	-1.49	0.139
Depression subscale (HADS-D)	3.49 ± 2.92	4.46 ± 3.74	-1.46	0.148
PHQ-9 Depression severity			4.97	0.083
No (0-4)	51 (82.26%)	53 (89.83%)		
Mild (5-9)	6 (9.68%)	6 (10.17%)		
Moderate (10-14)	5 (8.06%)	0		
EORCT QLQ-C30				
Functional Scales				
Physical Functioning	65.57 ± 23.42	74.12 ± 25.43	-1.89	0.061
Role Functioning	44.86 ± 32.55	58.78 ± 36.32	-2.18	0.031
Emotional Functioning	93.53 ± 8.89	96.07 ± 6.49	-1.76	0.080
Cognitive Functioning	84.78 ± 14.63	91.22 ± 13.22	-2.50	0.014
Social Functioning	42.84 ± 30.20	59.02 ± 33.17	-2.76	0.007
Global Health	64.40 ± 11.26	64.34 ± 14.51	0.02	0.981
Symptom scales				
Fatigue	21.66 ± 15.50	17.37 ± 17.80	1.39	0.168
Nausea and Vomiting	6.62 ± 15.29	5.93 ± 17.98	0.22	0.824
Pain	56.84 ± 24.85	41.22 ± 30.44	3.04	0.003
Dyspnoea	15.41 ± 19.90	11.78 ± 18.24	1.03	0.305
Insomnia	20.60 ± 23.23	15.75 ± 21.74	1.17	0.245
Appetite Loss	37.33 ± 31.98	27.02 ± 30.63	1.78	0.078
Constipation	21.66 ± 18.16	20.76 ± 22.18	0.24	0.812
Diarrhoea	0.57 ± 4.33	1.71 ± 7.37	-1.01	0.313
Financial Difficulties	57.98 ± 31.08	42.88 ± 32.30	2.58	0.011

Data are means ± SD or n (%). Percentages might not total 100% because of rounding. PG-SGA, Patient-Generated Subjective Global Assessment; NRS, Numerical Rating Scale; HADS, Hospital Anxiety and Depression Scale; PHQ-9, Patient Health Questionnaire-9; EORCT QLQ-C30, European Organization for Reasearch and Treatment of Cancer Quality-of-Life Questionnaire Core 30.

The safety profile of JK5G postbiotics in lung cancer patients mirrored that of the overall study population. The type, severity, and frequency of AEs were comparable between the JK5G and control groups. All AEs were manageable and did not affect treatment adherence (**Table 6**). There was no increase in opioid-related adverse events, and no cases of suicide attempts or opioid misuse were reported.

**Table 6:** Frequency of opioid-related adverse events in patients with lung cancer: subgroup analysis.

Adverse events	Control group (n=62)	JK5G group (n=59)	χ^2^	*P*
Total events	13 (20.00%)	10 (17.57%)	0.32	0.573
Constipation	11 (17.33%)	9 (16.22%)	0.14	0.713
Nausea	1 (1.33%)	1 (1.35%)	<0.01	0.972
Vomiting	1 (1.33%)	0	0.96	0.327

Data are presented in n (%).

In summary, this subgroup analysis confirms that the efficacy of JK5G postbiotics in alleviating cancer-related pain and improving quality of life—now including a significant benefit in role functioning—is consistent and particularly evident in patients with lung cancer.

### 3.8 JK5G postbiotics modulate gut microbiota and systemic immunity in lung cancer patients

**Figure 4:**
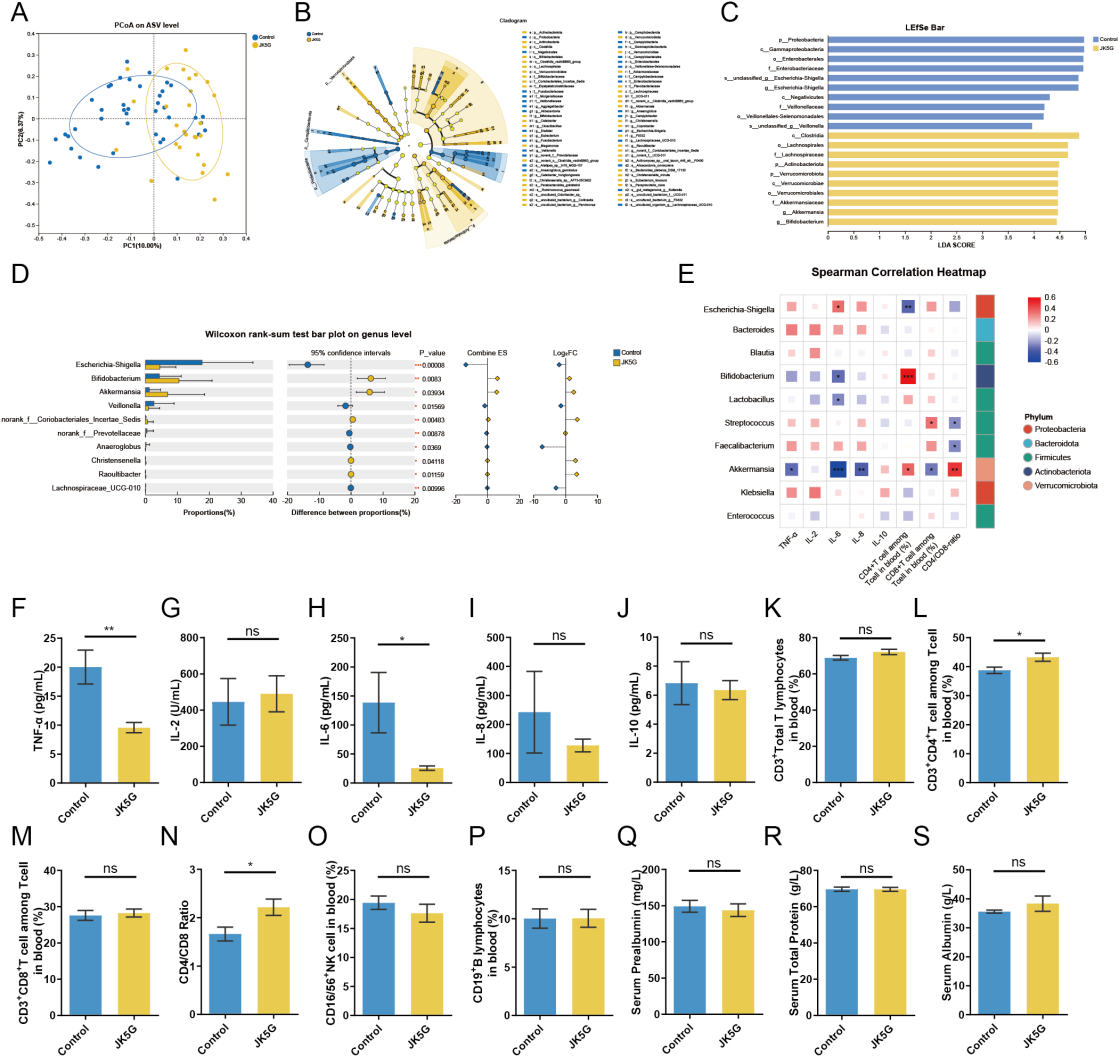
JK5G postbiotics modulate gut microbiota and systemic immunity in lung cancer patients. **(A)** Principal coordinate analysis (PCoA) based on unweighted UniFrac distance (ASV level). **(B)** Cladogram of gut microbiota (JK5G vs. control groups). **(C)** Linear Discriminant Analysis (LDA) scores from LEfSe showing biomarker taxa (LDA score > 2, *p* < 0.05 by Wilcoxon signed-rank test). **(D)** Wilcoxon rank-sum test bar plot on genus level of two groups. **(E)** Correlation heatmap between immune cells, cytokines, and bacterial genera (red: positive, blue: negative, white: no correlation). **(F–J)** Serum levels of TNF-α **(F)**, IL-2 **(G)**, IL-6 **(H)**, IL-8 **(I)**, and IL-10 **(J)** (JK5G vs. control). **(K–P)** Proportions of CD3^+^ T cells **(K)**, CD3^+^CD4^+^ T cells **(L)**, CD3^+^CD8^+^ T cells **(M)**, CD4^+^/CD8^+^ ratio **(N)**, CD16^+^/56^+^ NK cells **(O)**, and CD19^+^ B cells **(P)** in peripheral blood (JK5G vs. control). **(Q–S)** Nutrition-related indicators: serum prealbumin **(Q)**, total protein **(R)**, and albumin **(S)** (JK5G vs. control). Data in panels **(F–S)** were analyzed by Student’s t-test. *p < 0.05, **p < 0.01, ***p < 0.001, ns: not significant.

Building upon the efficacy and safety profile observed in the overall cohort and confirmed in the lung cancer subgroup, we further investigated the impact of JK5G postbiotics on gut microbial communities and systemic immune responses specifically in patients with lung cancer. PCoA analysis revealed that the compositional differences in gut microbiota between the JK5G and control groups were slightly more pronounced within the lung cancer subgroup compared to the overall cohort (**Figure 4A**). LEfSe and cladogram analyses indicated that JK5G intervention promoted the enrichment of beneficial bacteria, including the SCFA-producers *Bifidobacterium* and *Akkermansia muciniphila*, whereas the pathogenic *Escherichia-Shigella* was more abundant in the control group after treatment (**Figures 4B, C**). Genus-level comparisons between the control and JK5G groups confirmed that these differences were statistically significant (**Figure 4D**).

Correlation analysis further linked these microbial shifts to systemic immune and inflammatory parameters (**Figure 4E**). In the lung cancer subgroup, *Akkermansia muciniphila* abundance correlated positively with CD3^+^CD4^+^ T−cell percentage and the CD4^+^/CD8^+^ ratio, and negatively with serum TNF−α, IL−6, IL−8 levels and CD3^+^CD8^+^ T−cell percentage. *Bifidobacterium* showed a positive correlation with CD3^+^CD4^+^ T cells and a negative correlation with IL−6. Conversely, *Escherichia-Shigella* correlated negatively with CD3^+^CD4^+^ T cells and positively with IL−6, while *Lactobacillus* was negatively associated with IL−6. These correlation patterns were largely consistent with the overall cohort, though some associations appeared more pronounced in the lung cancer subgroup. Consistent with the microbial and correlative findings, JK5G administration in lung cancer patients significantly reduced serum levels of the pro−inflammatory cytokines TNF−α (**Figures 4F**) and IL−6 (**Figure 4H**), and increased the percentage of CD3^+^CD4^+^ T cells as well as the CD4^+^/CD8^+^ ratio (**Figures 4L, N**) compared to controls. No significant changes were observed in other immune subsets or nutritional markers (**Figures 4F–S**). These coordinated changes indicate that JK5G postbiotics can positively regulate both the gut ecosystem and the host immune-inflammatory state specifically in patients with lung cancer.

### 3.9 JK5G postbiotics affects fecal metabolic profiles in patients with moderate to severe cancer pain

**Figure 5:**
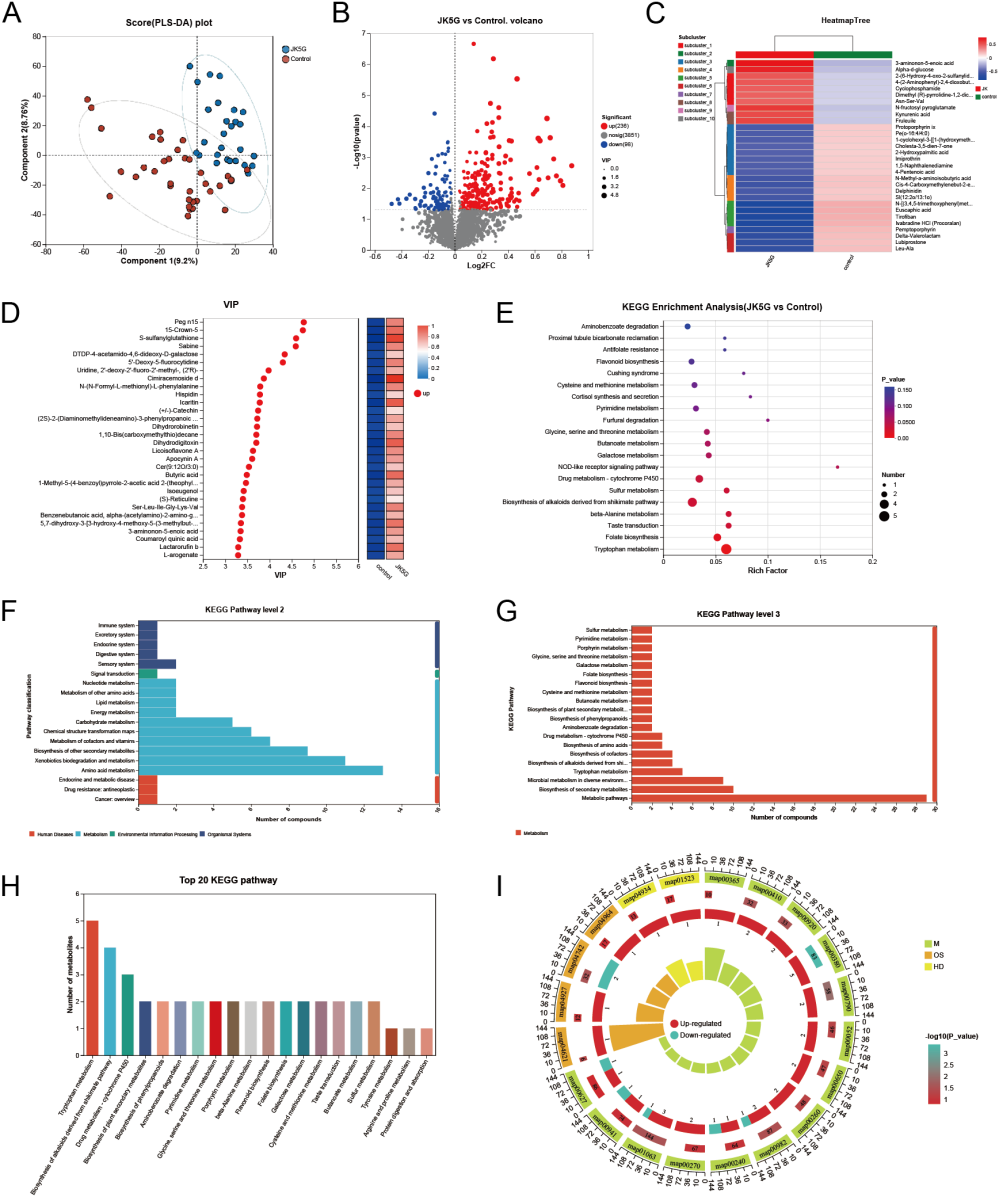
Effects of JK5G postbiotics on fecal metabolomics in cancer pain patients. **(A)** PLS-DA score plot between groups. **(B)** Volcano plot of differential metabolites (red: up-regulated; blue: down-regulated). **(C)** Heatmap of differential metabolites (color indicates relative expression: red for high, blue for low). **(D)** Variable importance in projection (VIP) scores of metabolites (VIP > 1); heatmap shows abundance ratio in JK5G vs. control (red: high, blue: low). VIP scores based on PLS-DA model. **(E)** KEGG pathway analysis: circle size = number of metabolites, color = *P* value. **(F)** KEGG pathway Level 2, **(G)** Level 3, **(H)** Top 20 KEGG pathways, and **(I)** KEGG multi-dimensional enrichment diagram.

Fecal samples were analyzed using UHPLC-MS/MS to investigate whether JK5G postbiotics can affect cancer pain by modulating intestinal flora and consequently altering fecal metabolites. PLS-DA was utilized to identify differential metabolites between groups (23). As shown in **Figure 5A**, significant differences were observed between the groups. However, some overlap was observed in the transitional zone, indicating partial similarity between groups. Significantly altered metabolites were identified using databases HMDB and Metlin based on the following criteria: 1) VIP > 1 by OPLS-DA model and 2) *p* < 0.05 by Student’s t-test. In the volcano plot, red dots represent increased expression and blue dots represent decreased expression (**Figure 5B**). Volcano plot analysis of fecal metabolomics revealed that JK5G postbiotics significantly altered the metabolite profiles of cancer pain patients compared to controls, with 236 metabolites upregulated and 98 downregulated (**Figure 5B**). To visualize the differences in the fecal metabolome associated with or without JK5G postbiotics treatment, we performed hierarchical clustering analysis (HCA) with a heatmap. Ten distinct clusters were formed among these differential metabolites. Significant increases in tryptophan-derived metabolites, including kynurenic acid and 4-(2-aminophenyl)-2,4-dioxobutanoic acid, were observed in the JK5G postbiotics group (**Figure 5C**). The VIP score derived from the PLS-DA model reflects the potential of the metabolite to serve as a biomarker (**Figure 5D**), and variables with VIP scores greater than 1.5 were considered important for the classification model. Thirty metabolites had VIP scores > 1.5, including butyric acid (VIP = 3.49). The key metabolites in cancer pain patients were identified via metabolic pathway analysis (**Figure 5E**). Eight signaling pathways were significantly affected between the Control and JK5G postbiotics groups (*p* < 0.05). The eight pathways were involved in tryptophan metabolism, folate biosynthesis, taste transduction pathway, β-Alanine metabolism, biosynthesis of alkaloids derived from the shikimate pathway, sulfur metabolism, drug metabolism-cytochrome P450, and NOD-like receptor signaling pathway. The tryptophan metabolism pathway was the most significantly affected after JK5G treatment. Similarly, both the KEGG pathway analysis of differentially expressed metabolites and the list of top 20 KEGG pathways identified tryptophan metabolism and butanoate metabolism as the most significant and potentially valuable pathways for further investigation (**Figures 5F–I**). These findings indicate that tryptophan metabolism and butanoate metabolism may plays a significant role in JK5G-mediated amelioration of moderate-to-severe cancer pain.

### 3.10 Identification of metabolome biomarkers via machine learning approaches

**Figure 6:**
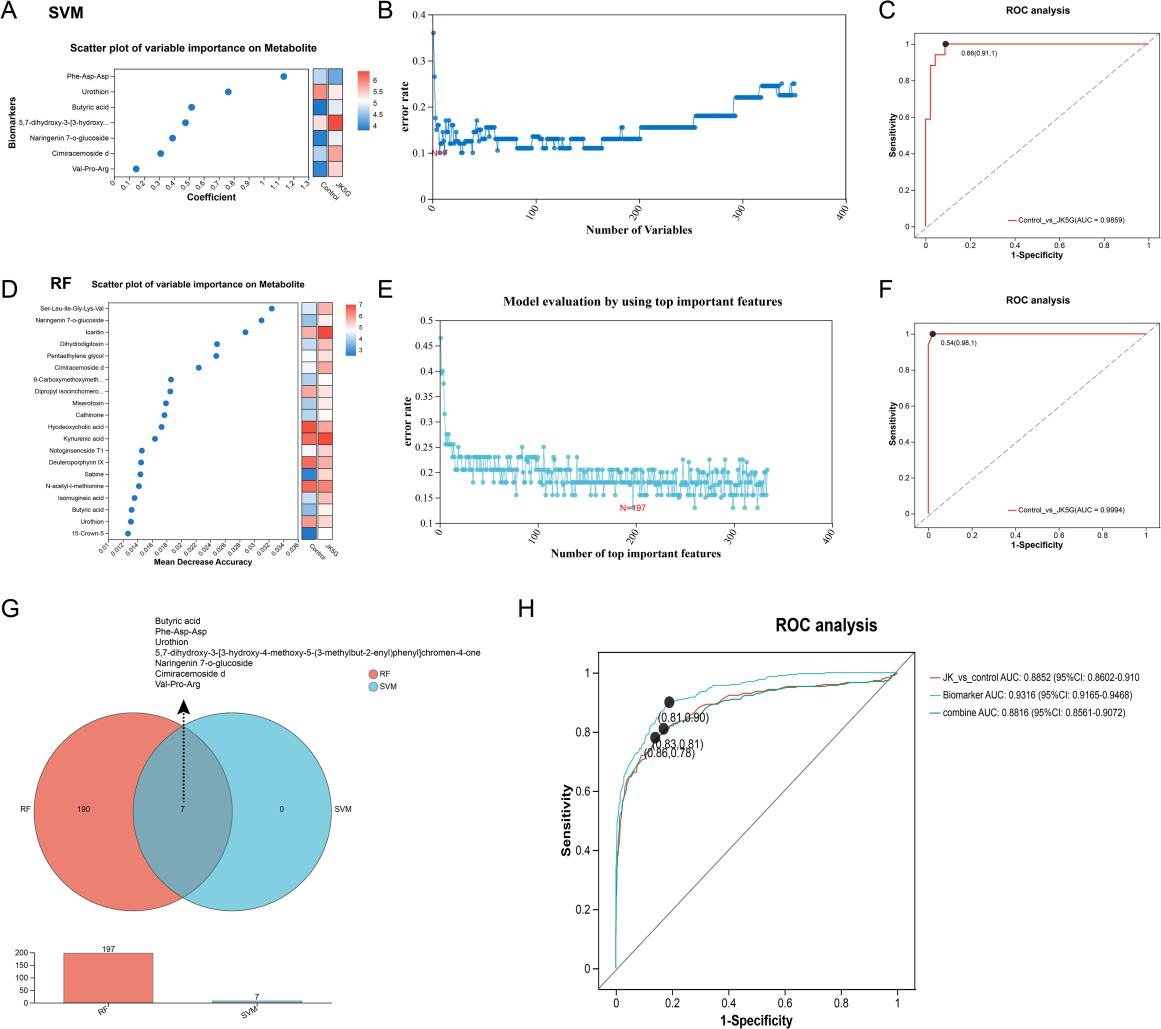
Development and validation of machine learning models for metabolome biomarkers. Two machine learning algorithms used: **(A–C)** Support Vector Machine (SVM) and **(D–F)** Random Forest (RF) to identify unique biomarkers. **(A, D)** Metabolite importance ranking (y-axis: importance score; x-axis: importance measure divided by standard deviation). **(B, E)** Feature selection using Recursive Feature Elimination with Cross-Validation (RFECV) to determine the optimal subset of metabolites. **(C, F)** ROC curves demonstrating model performance, with AUC values indicating predictive accuracy. **(G)** Venn analysis for selecting common biomarker variables and creating a common set. **(H)** ROC curve analysis evaluating diagnostic performance of biomarker-based models.

To screen for exploratory biomarkers within the metabolic profile, we constructed classifiers using both SVM and RF algorithms. Feature importance ranking derived from the SVM model highlighted key metabolites, including Phe-Asp-As, urothion, and butyric acid, among others (**Figure 6A**). The SVM model identified seven metabolic biomarkers (**Figure 6B**), which demonstrated excellent discriminatory power with an AUC of 0.9859 in the ROC analysis (**Figure 6C**). Similarly, the RF approach selected 197 metabolites (**Figure 6E**), among which kynurenic acid and butyric acid were ranked within the top 20 most important features (**Figure 6D**). The RF classifier also exhibited outstanding performance, achieving an AUC of 0.9994 (**Figure 6F**). By examining the intersection of biomarkers selected by both models using a Venn diagram (**Figure 6G**), we identified seven common metabolites: butyric acid, Phe-Asp-Asp, urothion, 5,7-dihydroxy-3-[3-hydroxy-4-methoxy-5-(3-methylbut-2-enyl)phenyl]chromen-4-one, Naringenin 7-O-glucoside, cimiracemoside D, and Val-Pro-Arg. Subsequent ROC analysis using these seven biomarkers yielded an AUC of 0.9316 (**Figure 6H**), outperforming the classifier built with all 334 differentially expressed metabolites between the JK5G and control groups (AUC = 0.8852). This result underscores the potential of these metabolites, particularly butyric acid, as robust exploratory biomarkers associated with the intervention.

### 3.11 Metabolite set enrichment analysis of fecal metabolomics

Conventional enrichment analysis typically focuses on metabolites that are significantly upregulated or downregulated, often overlooking those with low abundance yet critical biological functions. In contrast, metabolite set enrichment analysis (MSEA) incorporates predefined sets of metabolites associated with specific biological processes, thereby highlighting meaningful differences through integrated pathway-level analysis.

Using MSEA based on over-representation analysis (ORA), we categorized 334 differentially expressed fecal metabolites from patients with moderate to severe cancer pain following JK5G postbiotic intervention (**Supplementary Figure 6**). The analysis revealed significant enrichment among metabolite sets related to chemical structure, with the top 20 pathways displayed according to enrichment ratio (**Supplementary Figure 6A**). Highly enriched pathways included “Catecholamine Biosynthesis”, “Tryptophan Metabolism”, “Fatty Acid Biosynthesis”, and “Butyrate Metabolism”. Consistent with these findings, enrichment network analysis also showed pronounced clustering within “Tryptophan Metabolism”, “Fatty Acid Biosynthesis”, and “Butyrate Metabolism” (**Supplementary Figure 6B**).

These results corroborate earlier metabolomic and biomarker findings, suggesting that “Tryptophan Metabolism” and “Butyrate Metabolism” may represent key mechanistic pathways involved in the response to JK5G postbiotics.

### 3.12 Correlation analysis between gut microbiota, key physiological biochemistry factor and metabolites

In order to explore the relationship among the composition of gut microbiota, key physiological biochemical factors, and metabolites, spearman correlation analysis was performed (**Supplementary Figure 7**). Kynurenic acid showed a negative correlation with the percentage of CD16/56^+^ NK cells in blood, while 4-(2-aminophenyl)-2,4-dioxobutanoic acid was positively correlated with the percentage of CD4^+^ T cells among T cells in blood and negatively correlated with serum TNF-α (**Supplementary Figure 7A**). Specifically, there was a significant correlation between certain gut bacterial strains and fecal metabolites. Among these, kynurenic acid positively correlated with *Lactobacillus* but negatively correlated with *Lachnoclostridium* and *Anaerostipes* (**Supplementary Figure 7B**). Three bacterial strains—*A. muciniphila*, *Bifidobacterium*, and *Lactobacillus*—had positive correlations with 4-(2-aminophenyl)-2,4-dioxobutanoic acid (**Supplementary Figure 7B**). These findings suggest that JK5G may be associated with modulation of the gut microbiota and fecal metabolomic profiles, which could potentially influence systemic immune and metabolic states, and thereby have indirect implications for the tumor microenvironment. This modulation may contribute to enhanced analgesic efficacy and optimized pain management processes in cancer pain.

### 3.13 Microbial contribution to metabolite variance based on MIMOSA2

**Figure 7:**
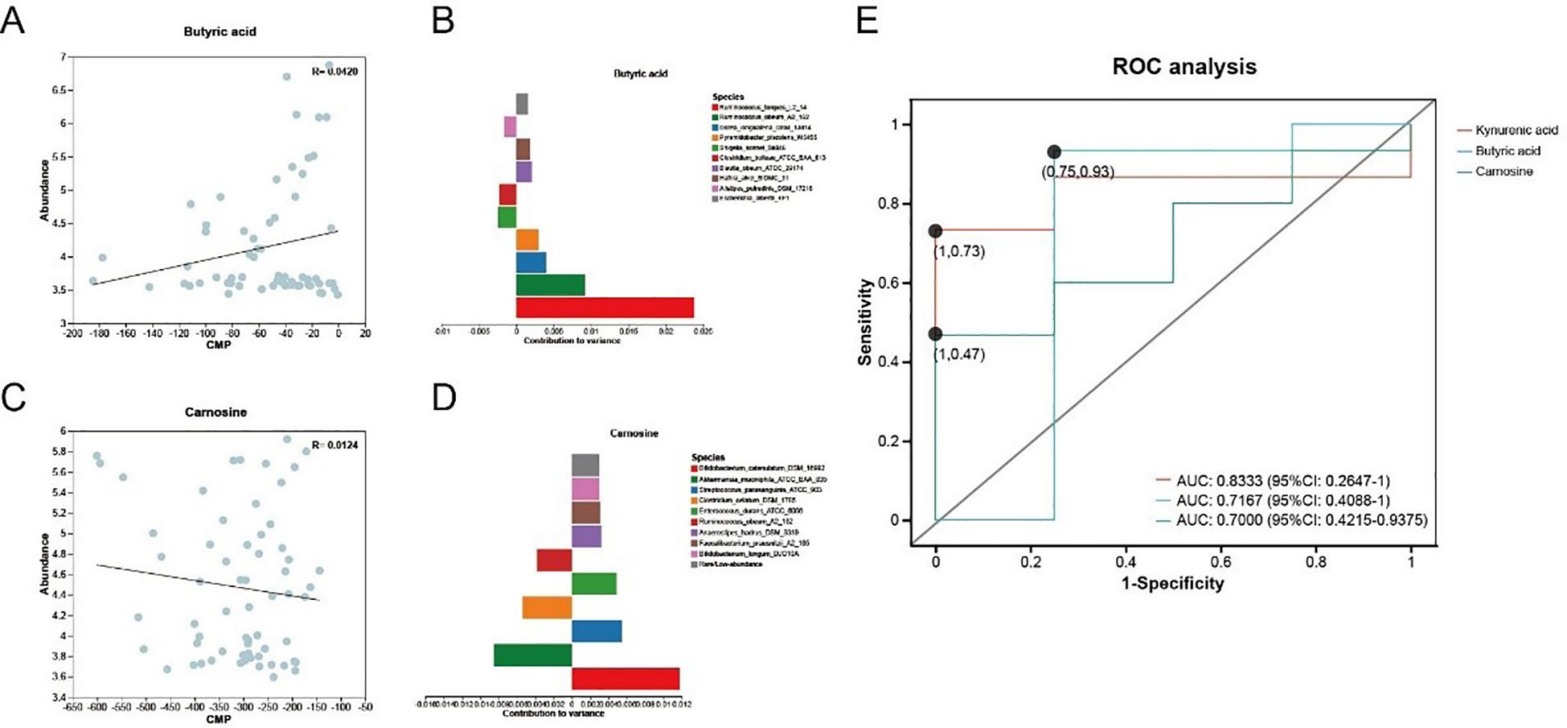
Microbial contribution to metabolite variance analyzed by MIMOSA2. MIMOSA2 analysis of microbial metabolic contributions to butyric acid **(A, B)** and carnosine **(C, D)**. **(A, C)** Regression plots of community-level metabolic potential (CMP) scores vs metabolite concentrations (R² = microbial contribution to variance; model *p* < 0.1). **(B, D)** Taxonomic contribution profiles: microbial species (Y-axis) ranked by contribution values (X-axis) (positive/negative values indicate microbial promotion/consumption). **(E)** ROC curve analysis of metabolite-based biomarker diagnostic performance.

MIMOSA2 employed genomic and metabolic reference databases to construct a community-wide metabolic model based on microbiome data, which was subsequently used to predict differences in metabolite levels among samples. These predictions were compared with metabolomics data to identify exploratory biomarker microbiome-controlled metabolites and to determine the taxonomic contributors to metabolite variation. Using the CMP scoring model, metabolites with accurate predictions were identified between the JK5G intervention and control groups. The results indicated that the gut microbial community contributed to changes in the abundance of two metabolites: butyric acid and carnosine (**Figures 7A, C**). As shown in **Figure 7A**, a correlation was observed for butyric acid (R = 0.0420). The contribution bar plot (**Figure 7B**) revealed the top five taxonomic contributors positively associated with butyric acid, namely *Ruminococcus torques*, *Ruminococcus obeum*, *Dorea longicatena*, *Pyramidobacter piscolens*, and *Blautia obeum*. Similarly, a modest correlation was found for carnosine (R = 0.0124, **Figure 7C**). *Bifidobacterium catenulatum* was identified as the strongest positive contributor for carnosine, whereas *Akkermansia muciniphila* exhibited the most pronounced negative contribution (**Figure 7D**). These findings align with previous diversity, metabolomic differential, and biomarker screening analyses, suggesting that butyric acid may play an important functional role in the observed metabolic profile. ROC analysis of key metabolite biomarkers between groups (**Figure 7E**) yielded AUC values of 0.8333 for kynurenic acid, 0.7167 for butyric acid, and 0.7000 for carnosine. Although these values indicate reasonably good discriminatory performance, they are slightly lower than the AUC of 0.9316 achieved by the seven-biomarker panel identified in prior screening (**Figure 6H**). These results suggest that while the metabolites identified through MIMOSA2 and differential analysis exhibit moderate predictive ability, the seven exploratory biomarkers set demonstrates superior performance, potentially due to synergistic effects between a broader range of metabolites. Further validation in independent cohorts and investigation into the mechanistic roles of these microbial contributors may strengthen the biological interpretation and translational potential of these findings.

## 4 Discussion

Cancer-related pain, particularly in its moderate to severe forms, remains a profound clinical challenge that significantly compromises patient QoL and treatment outcomes (1). While opioid analgesics are the cornerstone of management, their efficacy is often limited by inadequate pain relief, significant adverse effects, and the risk of tolerance or dependency (4). This underscores an urgent need for novel adjunctive strategies that can enhance analgesia while mitigating the burdens associated with conventional opioid therapy.

To address this need, we conducted a randomized, double-blind, placebo-controlled trial to evaluate the adjunctive effect of JK5G postbiotics—a preparation of inactivated lactic acid bacteria and their metabolites—in patients with moderate to severe cancer pain receiving patient-controlled subcutaneous analgesia. Our primary finding is that JK5G supplementation was associated with significant reductions in pain intensity and improvements in QoL compared to placebo. Furthermore, through a multi-omics approach, we observed that these clinical benefits were paralleled by modulation of the gut microbiota, fecal metabolome, and systemic immune markers. Although this study design identifies associations rather than proven causality, the concurrent changes across the gut-microbiome-immune axis provide a compelling framework to discuss potential mechanisms by which targeting the gut ecosystem may influence cancer pain perception.

The validity of these mechanistic explorations is strengthened by the rigorous matching of baseline characteristics between groups, a methodological refinement over some prior studies where heterogeneous patient profiles could confound the interpretation of microbiome-related interventions (24, 25). This stringent approach, consistently applied across the overall cohort and the predominant lung cancer subgroup, allows us to more reliably attribute the observed phenotypic and omics changes—including the pronounced benefits in lung cancer patients—to the JK5G intervention itself.

Delving into the therapeutic scheme, the addition of JK5G postbiotics to standardized PCSA analgesia represents a mechanistically distinct multimodal pain management strategy. Prior clinical investigations into cancer pain have largely focused on the opioid titration, the adjunctive use of anti-neuropathic medications, or the implementation of invasive interventions, often with limited or short-term improvements in multidimensional QoL (26). By contrast, emerging evidence underscores the intricate interplay between gut microbiota, immune modulation, and neuroinflammatory signaling pathways. This interplay shapes both nociceptive processing and psychosocial outcomes in oncology populations (27, 28). The present findings, which demonstrate significant benefits in cognitive and social function as well as in alleviating patients’ economic distress, align with the hypothesis that targeted microbiome-based interventions may exert systemic effects extending beyond conventional analgesia. This is partly supported by multi-omics studies linking microbial metabolites—such as SCFA and tryptophan catabolites—to the modulation of central and peripheral sensitization and affective pain components (25, 29). Therefore, the observed multidimensional improvement in JK5G recipients suggests that postbiotics may influence pain perception and patient-reported outcomes through both direct neuroimmune crosstalk and indirect psychosocial mechanisms.

The observed improvement in multidimensional QoL—particularly within the social and cognitive domains—in the JK5G group suggests a therapeutic mechanism that extends beyond direct analgesia. This is significant because conventional opioid-based regimens, even when optimized, often fail to fully alleviate pain-related interference and impairment in these higher-order functions, likely due to unaddressed neuroinflammation and dysfunctional gut-brain-immune communication (30). Our findings align with emerging evidence that specific microbial taxa are crucial regulators of affective and cognitive dimensions of pain, in part through modulating neuroactive compound synthesis and blood-brain barrier integrity (31, 32). Specifically, we demonstrate that JK5G postbiotics promote a restorative shift in the gut ecosystem, characterized by the enrichment of beneficial commensals such as *Akkermansia muciniphila* and *Bifidobacterium*, alongside the suppression of pathobionts like *Escherichia-Shigella*. This curated microbial environment is functionally poised to enhance the production of metabolites like butyrate and kynurenic acid, which are known to support intestinal barrier integrity and possess neuromodulatory properties. This study therefore provides evidence that JK5G postbiotics may alleviate cancer-related pain and improve QoL, with our data suggesting an association with favorable modifications to the gut-microbiome-immune axis.

These findings gain context when compared to established literature on opioid-induced dysregulation. For instance, chronic opioid use (e.g., oxycodone) is associated with gut dysbiosis and a sustained pro-inflammatory state marked by elevated IL-6 and TNF-α (9). In contrast, JK5G supplementation in our cohort was associated with an enriched abundance of beneficial taxa, an upregulation of metabolites including butyrate and kynurenic acid (differing from the pro-inflammatory lipid metabolite profile observed with morphine (29)), and a reduction in serum TNF-α alongside an increase in CD3^+^CD4^+^ T cells. Collectively, these patterns suggest that JK5G could modulate key biological pathways—microbial composition, host metabolism, and immune tone—that are adversely affected by opioids. This positions JK5G as a candidate adjunct therapy worthy of further investigation to clarify its mechanistic role and clinical utility in managing cancer-related pain.

Regarding intervention safety, the mild adverse event profile observed across groups, which is comparable to prior research on postbiotic and probiotic preparations in oncology, supports the use of inactivated microbial components to minimize the risk of pathogen translocation, uncontrolled immune activation, or metabolic perturbations, even in immunocompromised populations (12, 33). Importantly, the absence of increased opioid misuse or psychiatric sequelae aligns with the hypothesized mechanism whereby gut-targeted interventions reinforce mucosal integrity and reduce systemic exposure to inflammatory mediators, thereby mitigating opioid-related adverse effects such as constipation and neuropsychiatric symptoms (24). This is further substantiated by reports indicating that disruptions in the gut microbiota can exacerbate these opioid-induced complications, suggesting a potential role for postbiotics in offsetting them (34). Consequently, the findings affirm that integrating JK5G postbiotics into cancer pain management neither elevates overall risk nor introduces new safety concerns, supporting its feasibility for broader clinical application.

The integration of multi-omics data further substantiates the mechanistic link between JK5G and immune modulation. Specifically, we demonstrate that JK5G postbiotics significantly modify the gut microbiome, leading to an enrichment of beneficial taxa, including SCFA-producing bacteria such as *Bifidobacterium* and *Akkermansia muciniphila*. This modulation, achieved within a standardized nutritional framework, aligns with the principles of immunonutrition by targeting the gut-immune axis. The associated increase in SCFA production is known to contribute to improved intestinal barrier function and the suppression of pro-inflammatory cytokines (18), thereby highlighting the role of JK5G in regulating T cell-mediated immunity and overall immune responses. This immunomodulatory effect provides a plausible biological basis for the observed reduction in systemic inflammation and the associated improvements in pain and QoL.

Furthermore, metabolomic analysis revealed significant alterations in key metabolic pathways, particularly tryptophan metabolism. The observed elevation of kynurenic acid—a tryptophan-derived metabolite with established neuroprotective and pain-modulating properties—suggests a plausible mechanism through which JK5G postbiotics may exert analgesic effects. Kynurenic acid is known to modulate central glutamate signaling and has been shown to alleviate pain-related behaviors in preclinical models (35). Concurrent reductions in systemic inflammatory markers such as TNF-α, alongside shifts in fecal metabolite profiles, indicate a broader reprogramming of host metabolism that extends to pathways like butanoate metabolism. These pathways are critically involved in neuroimmune crosstalk and may influence the psychological and neurological symptoms frequently reported by cancer patients (25, 31).

Collectively, these multi-omics findings reinforce the gut-immune-brain axis hypothesis, wherein microbial metabolites can modulate peripheral and central pain-processing circuits. They underscore the value of integrating immune and metabolic endpoints into the evaluation of microbiome-targeted interventions. This approach advances our understanding of how specific microbial metabolites, including kynurenic acid, may contribute to developing more effective pain management strategies (36, 37). The integration of microbiome and metabolomic data, particularly through machine learning-identified biomarkers, also highlights the potential for personalized therapeutic approaches in cancer pain.

Finally, a key methodological strength of this study is the rigorous exclusion of patients with recent exposure to antibiotics, immunosuppressants, or other microbiota-modulating treatments—a common confounder that has often obscured causal inference in prior research (24, 34). Such exposures can induce prolonged shifts in microbial ecology and immune status, thereby potentially masking or mimicking intervention effects (25). By controlling for these variables, our design provides a clearer attribution of the observed changes to the direct impact of JK5G on the host-microbiome-immune-metabolic network. This methodological rigor supports more definitive mechanistic interpretation and establishes a stronger foundation for future translational studies on targeted postbiotics in cancer pain management.

This study presents several notable limitations that merit careful consideration. First, the single-center design and the recruitment of participants exclusively from Chongqing University Cancer Hospital may restrict the external validity of our findings. Furthermore, our cohort predominantly consisted of patients with lung cancer (approximately 80%), with a minority of other cancer types. This composition may limit the generalizability of our conclusions to a broader oncology population, as pain mechanisms, treatment responses, and gut microbiome profiles can vary significantly across different cancer types. The homogeneity of the study population, shaped by specific regional, dietary, and healthcare practices, may not reflect the broader, more heterogeneous cancer patient populations found in different geographic or ethnic settings. Second, the taxonomic and functional resolution of our microbial analysis is constrained by the use of 16S rRNA gene sequencing targeting the V3-V4 region. This method provides reliable identification primarily at the genus level, and any associated functional inferences are speculative and require validation through metagenomic, metatranscriptomic, or direct experimental approaches. Third, while our short-term safety assessment, including analysis of common tumor markers (CEA, CYFRA 21-1, CA125, CA19-9, NSE), showed no adverse signals, the 7-day intervention period precludes a definitive evaluation of the long-term impact of JK5G on tumor progression or dynamics. Furthermore, the relatively short intervention duration (7 days) also limits our ability to assess the long-term sustainability of JK5G’s effects on gut microbiota, immune responses, and metabolic profiles, leaving potential delayed benefits or adverse events uncharacterized.

In summary, our randomized, double-blind, placebo-controlled trial demonstrates that adjunctive JK5G postbiotics can significantly improve QoL and beneficially modulate gut microbiota, immune parameters, and fecal metabolomics profiles in patients with moderate to severe cancer pain receiving opioid-based analgesia. By integrating multi-omics approaches with comprehensive clinical phenotyping, this research emphasizes the translational potential of postbiotic interventions in supportive oncology. The identification of key microbial and metabolomic biomarkers further supports the role of JK5G as a promising adjunctive therapy for cancer pain management. Future research should focus on extending treatment duration and enrolling cohorts from multiple centers and diverse ethnic backgrounds. It should also aim to elucidate the mechanistic pathways linking gut microbiota alterations to pain modulation and systemic immune responses. Additionally, these findings pave the way for personalized postbiotic therapies in cancer pain management, warranting further exploration in larger, longitudinal clinical trials.

## Data Availability

The datasets presented in this study can be found in online repositories. The names of the repository/repositories and accession number(s) can be found below: The sequences reported in this paper have been deposited in the NCBI database (accession number PRJNA1285121) and National Genomics Data Center (PRJCA042538). Additional information required to reanalyse the data reported in this study is available from the corresponding author on reasonable request.

## Supplementary material

Supplementary Figure 1 Trial profile.

Supplementary Figure 2 **(A)** Rarefaction curves of sequencing. The α diversity of intestinal microbial compositions evaluated by Chao1 **(B)**, ACE **(C)**, and Shannon **(D)** indexes at baseline and after four treatment cycles. Data are represented as medians with interquartile ranges; ns: not significant.

Supplementary Figure 3 Bar chart of performance evaluation for four machine learning models in microbiome biomarker selection. **(A)** SVM, **(B)** RF, **(C)** LASSO, **(D)** Logistic Regression.

Supplementary Figure 4 Nutrition-related indicators Serum Total Protein **(A)**, Serum Albumin **(B)**, and Serum Prealbumin **(C)** in blood between two group. Student’s t-test, ns: not significant.

Supplementary Figure 5 Common tumor markers CEA **(A)**, CYFRA 21-1 **(B)**, CA125 **(C)**, CA199 **(D)**, and NSE **(E)** in blood between two groups. Student’s t-test, ns: not significant.

Supplementary Figure 6 Metabolite set enrichment analysis (MSEA) of fecal metabolomics in cancer pain patients. **(A)** Dot plot and **(B)** MSEA enrichment network analysis of metabolites affected by JK5G postbiotics. Analysis used Over Representation Analysis (ORA) approach referencing Small Molecule Pathway Database (SMPDB) to identify enriched pathways. Left: colors indicate *p*-value; dot size shows enrichment ratio (hits/expected). Right: correlations between key metabolites and pathways.

Supplementary Figure 7 Heatmap of Spearman’s correlation analysis among exploratory biomarker metabolites, key physiological and biochemical factors, and microbiota **(A)** Metabolite–physiological factor correlations. **(B)** Metabolite–microbiota correlations. Colors show correlation coefficients (red: positive; blue: negative). Statistical significance: **P* < 0.05, ***P* < 0.01, ****P* < 0.001.

Supplementary Material S1 Standardized Dietary and Lifestyle Management Protocol.
