# Supplementary material for: JK5G postbiotics modulate gut microbiota and metabolome to alleviate cancer-related pain: a randomized controlled trial with multi-omics integration
Source: Front Immunol. 2026 Mar 4;17:1764491. doi: 10.3389/fimmu.2026.1764491 (PMC12995801; doi:10.3389/fimmu.2026.1764491)
Supplement: Supplementary Figure 1 — Trial profile. [file DataSheet1.pdf]

JK5G Postbiotics Modulate Gut Microbiota and Metabolome to Alleviate Cancer-Related Pain: A Randomized Controlled Trial with Multi-Omics Integration

Mengting Chen[1], Junhui Zhang[2, 3], Hong Yang[2, 3], Lei Lei[2, 3], Liejun Yang[2, 3], Sixiong Wang[2, 3], Huiqing Yu[1, 2, 3#]

[1]Department of Clinical nutrition,Chongqing University Cancer Hospital, School of Medicine, Chongqing University, Chongqing, China.

[2]Department of Geriatric Oncology, Chongqing University Cancer Hospital, School of Medicine, Chongqing University, Chongqing, China.

[3] Department of Palliative care, Chongqing University Cancer Hospital, School of Medicine, Chongqing University, Chongqing, China.

[#]Correspondent author: Huiqing Yu

Chongqing University Cancer Hospital, No. 181 Hanyu Road, Shapingba District, Chongqing 400030, P. R. China.

E-mail: yhqdyx@cqu.edu.cn (HQY)

Appendices. Supplementary data

Supplementary Figure 1. Trial profile

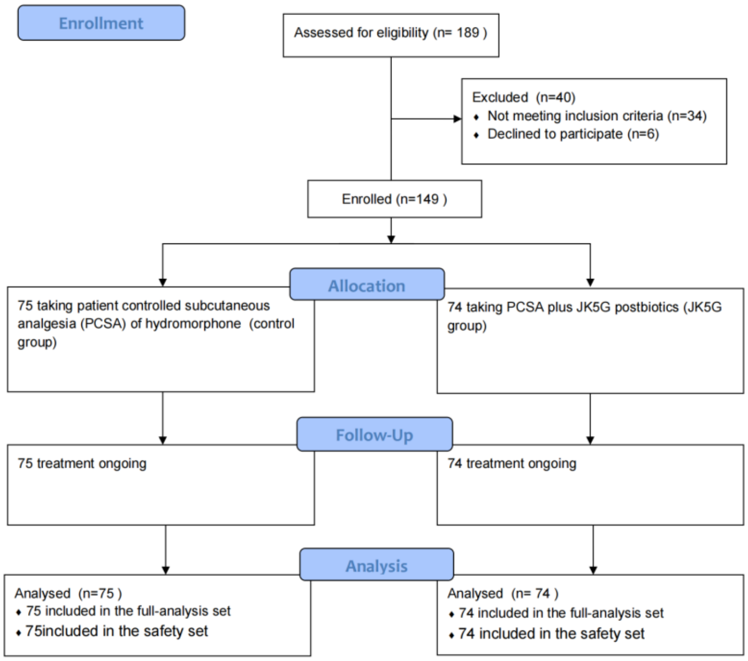

Supplementary Figure 2. (A) Rarefaction curves of sequencing. The α diversity of intestinal microbial compositions evaluated by Chao1 (B), ACE (C), and Shannon (D) indexes at baseline and after four treatment cycles. Data are represented as medians with interquartile ranges; ns: not significant.

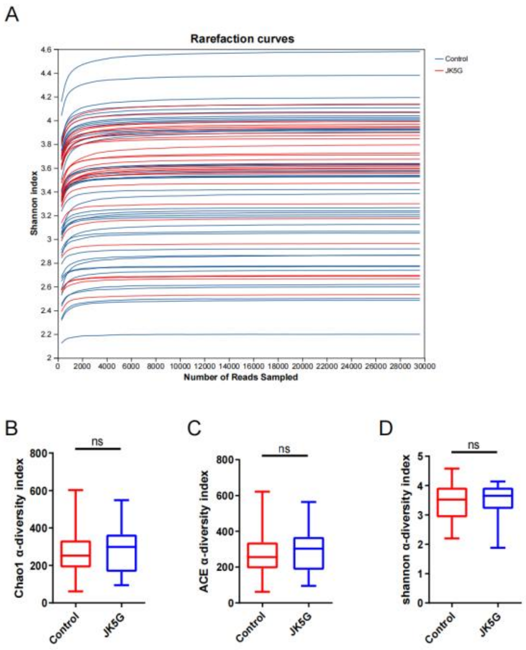

Supplementary Figure 3. Bar chart of performance evaluation for four machine learning models in microbiome biomarker selection. (A) SVM, (B) RF, (C) LASSO, (D) Logistic Regression.

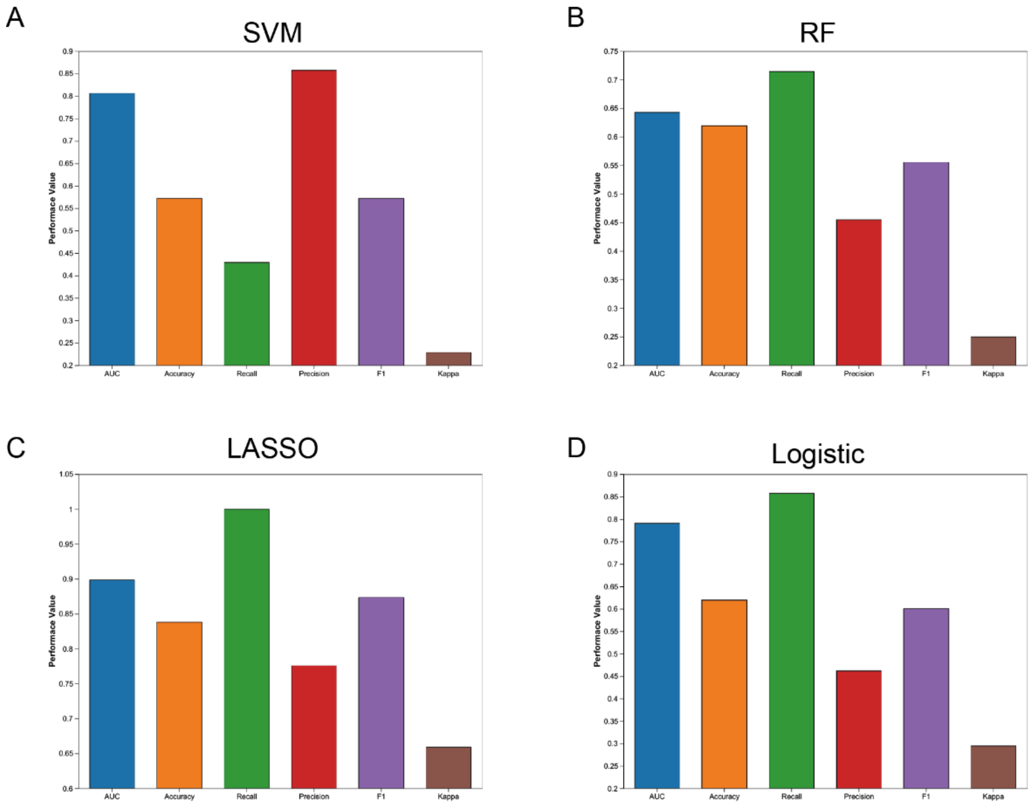

Supplementary Figure 4. Nutrition-related indicators Serum Total Protein (A), Serum Albumin (B), and Serum Prealbumin (C) in blood between two group. Student's t-test, ns: not significant.

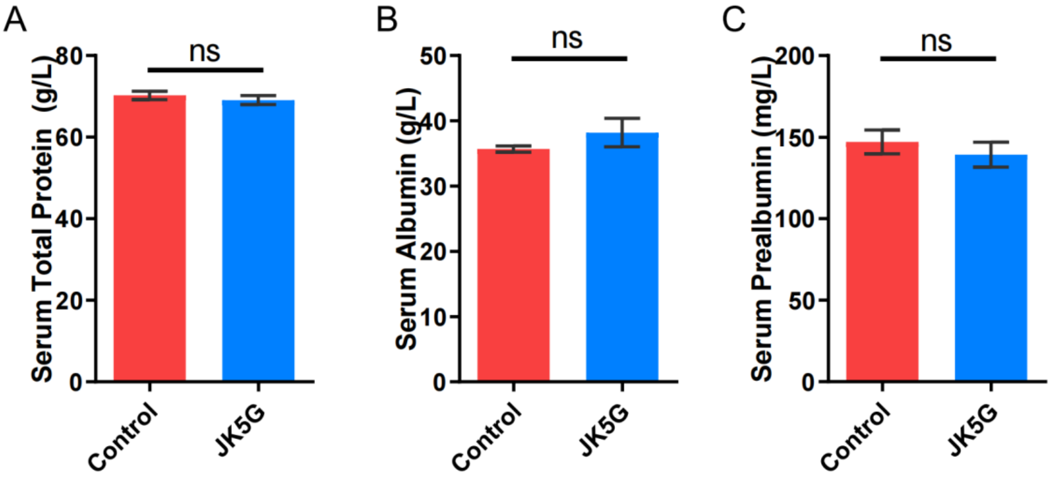

Supplementary Figure 5. Common tumor markers CEA (A), CYFRA 21-1 (B), CA125 (C), CA199 (D), and NSE (E) in blood between two groups. Student's t-test, ns: not significant.

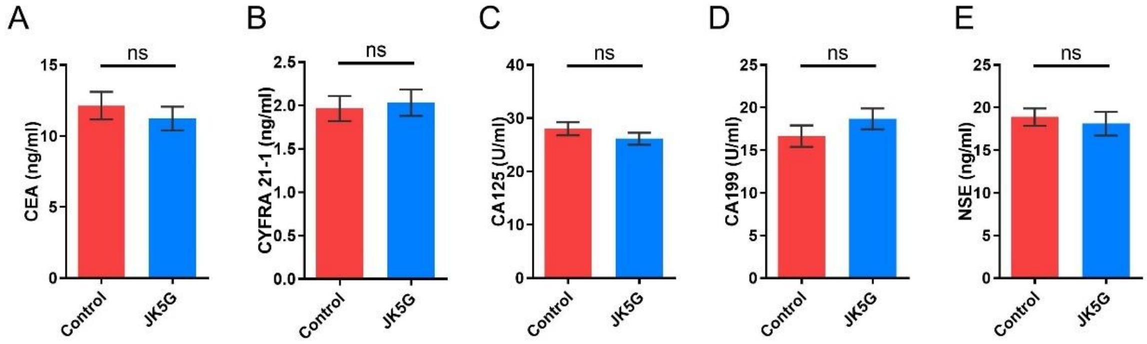

Supplementary Figure 6. Metabolite set enrichment analysis (MSEA) of fecal metabolomics in cancer pain patients. (A) Dot plot and (B) MSEA enrichment network analysis of metabolites affected by JK5G postbiotics. Analysis used Over Representation Analysis (ORA) approach referencing Small Molecule Pathway Database (SMPDB) to identify enriched pathways. Left: colors indicate *p*-value; dot size shows enrichment ratio (hits/expected). Right: correlations between key metabolites and pathways.

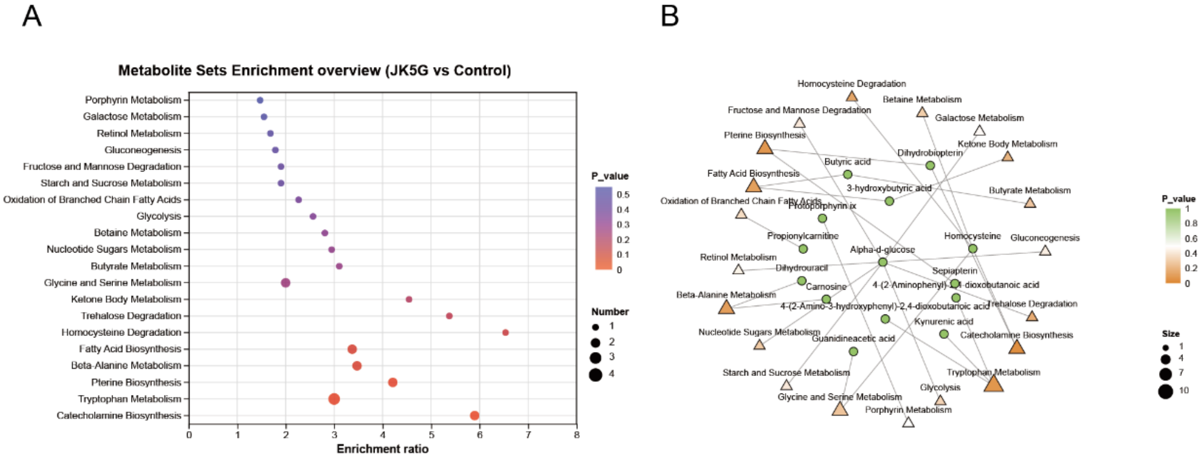

Supplementary Figure 7. Heatmap of Spearman's correlation analysis among exploratory biomarker metabolites, key physiological and biochemical factors, and microbiota (A) Metabolite–physiological factor correlations. (B) Metabolite–microbiota correlations. Colors show correlation coefficients (red: positive; blue: negative). Statistical significance: *$P < 0.05$, **$P < 0.01$, ***$P < 0.001$.

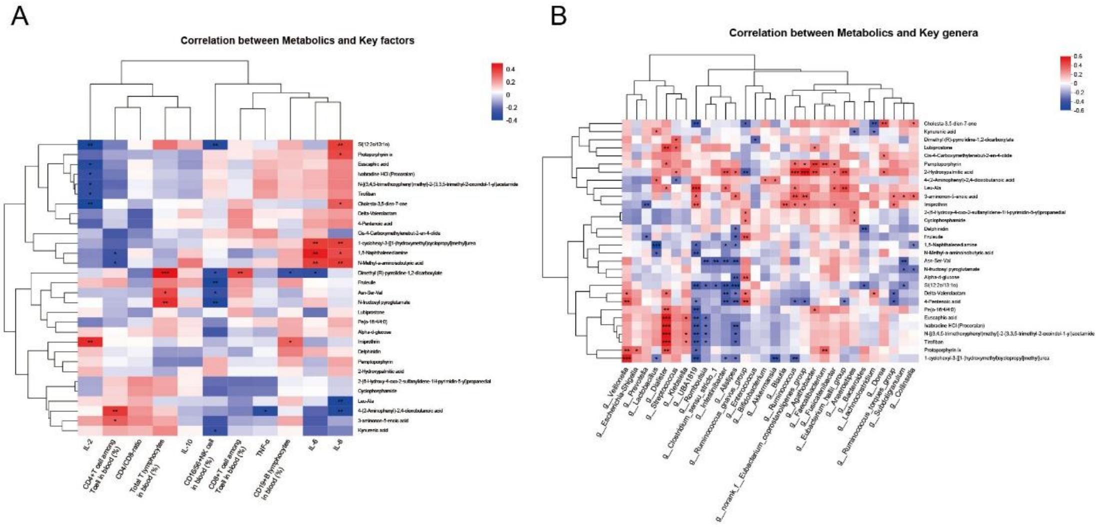

Supplementary Material S1: Standardized Dietary and Lifestyle Management Protocol

1. Dietary Management Protocol

1.1 Guiding Principles and Basis

Individualized dietary plans for all participants were formulated based on the Nutrition Guidelines for Chinese Cancer Patients (2022) and the Dietary Guidelines for Chinese Residents (2022). The core objective was to standardize energy and macronutrient intake to minimize dietary confounding effects on the gut microbiota.

1.2 Individualized Nutritional Assessment and Target Setting (Specific Procedures)

Assessment Tools:

Energy Requirements: Clinical dietitians calculated resting energy expenditure (REE) using the Harris-Benedict equation. Total daily energy expenditure (TDEE) was then determined by multiplying REE with stress factors (1.1-1.3) based on the patient's Eastern Cooperative Oncology Group (ECOG) Performance Status and disease state.

Body Composition: Bioelectrical impedance analysis (BIA) was used upon admission to measure body weight and fat-free mass (FFM), serving as the baseline for calculating protein requirements.

Specific Numerical Targets:

Energy Intake: 25-30 kcal/kg/day (based on actual body weight). For participants with a BMI < 18.5 kg/m², calculations were based on ideal body weight (height² × 21).

Protein Intake: 1.2-1.5 g/kg/day (based on actual body weight), with at least 50% derived from high-quality animal protein (e.g., eggs, lean meat, fish, whey protein).

Fat and Carbohydrates: Fat contributed 20-30% of total energy, and carbohydrates contributed 50-60%.

Dietary Fiber: A daily intake of ≥25 g was ensured, primarily from vegetables, fruits, and whole grains.

Fluids: Daily water intake was 30-40 mL/kg, excluding water content from food.

1.3 Meal Provision and Monitoring (Inpatients)

Meal Provision Mechanism: All meals for inpatients during the study period were uniformly prepared and delivered by the hospital's Department of Clinical Nutrition. The study utilized standardized therapeutic diet sets (categories A and B, corresponding to different energy levels).

Sample Meal Composition (Example for a ~1500 kcal set):

Breakfast (07:00): Whole-wheat steamed bun (80g), boiled egg (1 large, ~50g), unsweetened soy milk (200ml).

Morning Snack (10:00): Apple (150g).

Lunch (12:00): Steamed jasmine rice (cooked weight 150g, ~75g dry weight), steamed sea bass (edible portion 100g), stir-fried spinach with garlic (200g), cooking oil (10g).

Afternoon Snack (15:00): Walnuts (~15g) and a small orange (100g).

Dinner (18:00): Steamed mixed-grain rice (cooked weight 100g, ~50g dry weight of mixed grains), stir-fried chicken breast with broccoli (chicken breast 80g, broccoli 150g), cooking oil (10g).
Compliance Verification: Research nurses conducted daily visual inspections of meal tray returns and recorded the estimated percentage of food consumed on a Meal Intake Verification Form. The target was consumption of >90% of the provided portion.

1.4 Dietary Management for Ambulatory Patients
Toolkit: Patients were provided with a Home Dietary Guidance Manual, standardized food portion size guides, and a 3-Day Dietary Record Form.
Implementation and Feedback: Patients submitted one 3-Day Dietary Record Form per week (covering two weekdays and one weekend day). Dietitians reviewed these records via the 24-hour dietary recall method, providing feedback and adjustments via phone or follow-up visits to correct deviations.

2. Standardized Physical Activity Protocol
Prescription Principle: Activity levels were strictly graded according to ECOG Performance Status to avoid the influence of intense or irregular exercise [1].
Specific Protocol:
ECOG 0-1: Daily walking for 30 minutes (can be split into sessions), with a perceived exertion rate (RPE) target of 11-13 (fairly light).
ECOG 2: Daily bedside or indoor activities for 15-20 minutes, ensuring no shortness of breath or excessive fatigue.
Monitoring: Participants used a provided pedometer to record daily steps and completed a Daily Activity Log.

3. Quantitative Assessment of Protocol Adherence
Dietary Adherence Index (DAI): To quantitatively assess overall adherence to the prescribed dietary protocol, we calculated a composite Dietary Adherence Index (DAI). The formulation of this index was based on the established principle that adequate energy and protein intake are the primary and most critical goals in nutritional support for cancer patients [2, 3]. Accordingly, the DAI was calculated as follows:
DAI (%) = (Actual Total Energy Intake / Target Energy Intake) × 70 + (Actual Protein Intake / Target Protein Intake) × 30
A DAI score > 85% was pre-defined as indicating good overall dietary adherence, a threshold aligned with high adherence standards commonly used in clinical intervention studies [4].
Activity Adherence: Completion of the prescribed physical activity on ≥ 80% of monitored days was considered adherent.
Inter-group Comparison: The mean DAI was 88.3 ± 5.2% in the JK5G group (n=74) and 86.7 ± 6.1% in the control group (n=75) (p=0.32). Activity adherence rates were 86.7% and 85.1%,

respectively (*p*=0.78), confirming successful standardization of these key background factors between groups.

This rigorous, protocol-driven approach was implemented to minimize heterogeneity in dietary intake and physical activity—key modifiers of gut microbiota and metabolism—between the intervention (JK5G) and control groups. By standardizing these background factors, we aimed to isolate and more clearly attribute the observed changes in gut microbial composition, metabolome, and clinical outcomes to the JK5G postbiotic intervention itself, thereby strengthening the internal validity of our study conclusions.

Reference:

[1] Oncology, C. S. o. N., Regulation, K. L. o. S. A. f. M., CONG, M., SHI, H., Consensus of Chinese experts on exercise therapy for cancer patients. 2022, *52*, 1-16.
[2] Muscaritoli, M., Arends, J., Bachmann, P., Baracos, V., Barthelemy, N., Bertz, H., Bozzetti, F., Hütterer, E., Isenring, E., Kaasa, S., Krznaric, Z., Laird, B., Larsson, M., Laviano, A., Mühlebach, S., Oldervoll, L., Ravasco, P., Solheim, T. S., Strasser, F., de van der Schueren, M., Preiser, J. C., Bischoff, S. C., ESPEN practical guideline: Clinical Nutrition in cancer. *Clinical nutrition (Edinburgh, Scotland)* 2021, *40*, 2898-2913.
[3] Chinese Society of Clinical Oncology (CSCO). Guidelines of Chinese Society of Clinical Oncology nutrition in cancer patients. People's medical publishing house Press, Beijing, China, 2021.
[4] Osterberg, L., Blaschke, T., Adherence to medication. *The New England journal of medicine* 2005, *353*, 487-497.
